# Evolutionary Distribution Changes of Sichuan Golden Monkeys ( *Rhinopithecus roxellana* ) in the Quaternary

**DOI:** 10.1002/ece3.72036

**Published:** 2025-09-02

**Authors:** Hao Pan, Kang Huang, Jing Wang, Chengrui Song, Felix Guo, Xiao Zhang, Zhengfeng Hu, He Zhang, Kexin Ji, Shujun He, Songtao Guo, Dayong Li, Wenyong Tian, Haitao Zhao, Jianbing Yue, Ruliang Pan, Zhihua Zhou, Gang He, Baoguo Li

**Affiliations:** ^1^ Shaanxi Key Laboratory for Animal Conservation, College of Life Sciences Northwest University Xi'an China; ^2^ International Centre of Biodiversity and Primate Conservation Dali University Dali China; ^3^ Rangitoto College Auckland New Zealand; ^4^ Jiangxi Provincial Key Laboratory of Conservation Biology, College of Forestry Jiangxi Agricultural University Nanchang China; ^5^ Shaanxi Institute of Zoology Xi'an China; ^6^ Key Laboratory of Southwest China Wildlife Resources Conservation (Ministry of Education) Western China Normal University Nanchong China; ^7^ Management Bureau of Shaanxi Zhouzhi National Nature Reserve Xi'an China; ^8^ Wildlife Conservation Monitoring Center National Forestry and Grassland Administration Beijing China; ^9^ School of Human Sciences The University of Western Australia Perth Western Australia Australia; ^10^ College of Life Sciences Yanan University Yanan China

**Keywords:** animal habitat and ecological changes, historical changes in primate distribution in recent China, primate evolutionary development, Sichuan golden monkeys

## Abstract

Retrospective reconstruction of animals' evolutionary development and geographic changes in recent history, as well as monitoring contemporary conservation status, are critical to amending existing conservation strategies. This study presents an innovative effort, as demonstrated by the Sichuan golden monkeys ( *Rhinopithecus roxellana* ) in China. We studied their fossil distribution patterns during the Pleistocene, explored historical distribution changes over the last 400 years, and surveyed 27 nature reserves where they dwell. The results indicate that this species successfully dispersed from Southwest China to the rest of the mainland of East Asia along the Yangtze and Pearl Rivers, reaching Taiwan during the Pleistocene, which marked the broadest geographic distribution in its evolutionary history. Unfortunately, its population began to shrink during the Holocene, leaving it currently restricted to the provinces of Sichuan, Gansu, Shaanxi, and Hubei in China. Accordingly, we proposed four conservation strategies to amend its conservation status.

## Graphical abstract

Reconstruct the distribution profiles of the ancestor of *R. roxellana* during the Pleistocene. A Historical distribution alteration of *R. roxellana* , including the ancestor (fossils), Historical (Starting from 1100 BP), and current (field survey) data. The human active impact on the *R. roxellana* group wave, reflecting the impact of different ways, traditional use, war ruining, and modern exploitation of natural resources.

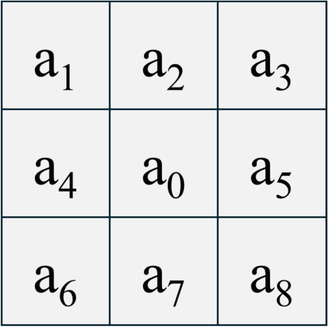

## 1 Introduction

China attracts significant global attention due to its grandeur of biodiversity and the prominent pressures on environmental protection and animal conservation (Dudgeon 2005; Li et al. 2022; Pan et al. 2016; Zhang et al. 2022). Evolutionarily, China has played a crucial role in faunal radiation in Southern, Southeastern, and Eastern Asia (Zhang et al. 2022). Unfortunately, a significant loss of biodiversity in the country began in the Early Holocene, which was accelerated in recent history, as evidenced by China's loss of more than 150 million hectares of forest and grassland area (Liu and Tian 2010). Additionally, China's remaining wildlife, particularly non‐human primates, has been severely impacted (Li et al. 2018).

To tackle such a difficult situation, the Chinese government has invested considerable effort in preservation since the beginning of this century, including expanding forest protection areas, promoting wildlife conservation, and emphasizing effective management (Delang 2016). Additionally, several non‐governmental ecological remediation projects, such as the Ant Forest project—a land restoration initiative by Alibaba—have been developed, making substantial progress in restoring degraded ecosystems (Zhang et al. 2020). Thus, by 2020, China had established 1,461,913 km^2^ of terrestrial protected area, covering over 90% of terrestrial ecosystems and 89% of nationally protected wildlife and plant species (He and Cliquet 2020).

However, China still faces enormous environmental and conservation pressures, particularly in reducing human impacts on protected areas (Zhao et al. 2021). Thus, in addition to investing financial resources and human capital, specific conservation strategies driven by scientific research achievements are critically needed.

The Sichuan golden monkey ( *Rhinopithecus roxellana* ), a member of the Colobinae subfamily within the Cercopithecidae family, is a first‐class protected animal species in China (Yu et al. 2022). It is classified as an endangered (EN) species on the IUCN Red List of Threatened Species in 2015 (Long and Richardson 2021).

Regarding its evolutionary development during the Pleistocene, a study outlined the general fossil distribution of the genus, including its four species in China. Still, it did not detail the specific dispersal process or distribution pattern of *R. roxellana* (Li et al. 2022). Such a distribution identification is necessary to explore the evolutionary trajectory of this species since the Pleistocene and project its prospective survival profiles, taking into account historical changes before 2000 and the current field distribution patterns. Besides providing scientific data and evidence for conservation, this endeavor also offers an engaging educational model of ecotourism, which aims to increase economic benefits for local villagers, reduce the extraction of natural resources, and raise public awareness about *R. roxellana* .

Therefore, the primary purposes of this study include: (1) reconstructing the evolutionary development of *R. roxellana* since the Pleistocene; (2) illustrating its historical distribution alterations before and after the 1800s; (3) presenting its demographic profiles in the current 27 nature reserves; and (4) ranking its population density regionally and exploring environmental and ecological factors impacting its conservation.

## 2 Materials and Methods

The data analyzed in this study include: (1) a fossil database containing the fossil‐bearing locations of *R. roxellana* in the Pleistocene, extracted from (Li et al. 2002; Zhang et al. 2022); (2) the historical distribution profiles of the species from 1304 to 2020 based on a broad literature review, and a search for local governmental annals and archives (Zhang et al. 2022); and (3) the records based on a field survey conducted from January 2018 to November 2019 in 27 national nature reserves across the Sichuan, Gansu, Shaanxi, and Hubei provinces in China.

### 2.1 Distribution of Fossil *R. roxellana*

**FIGURE 1:**
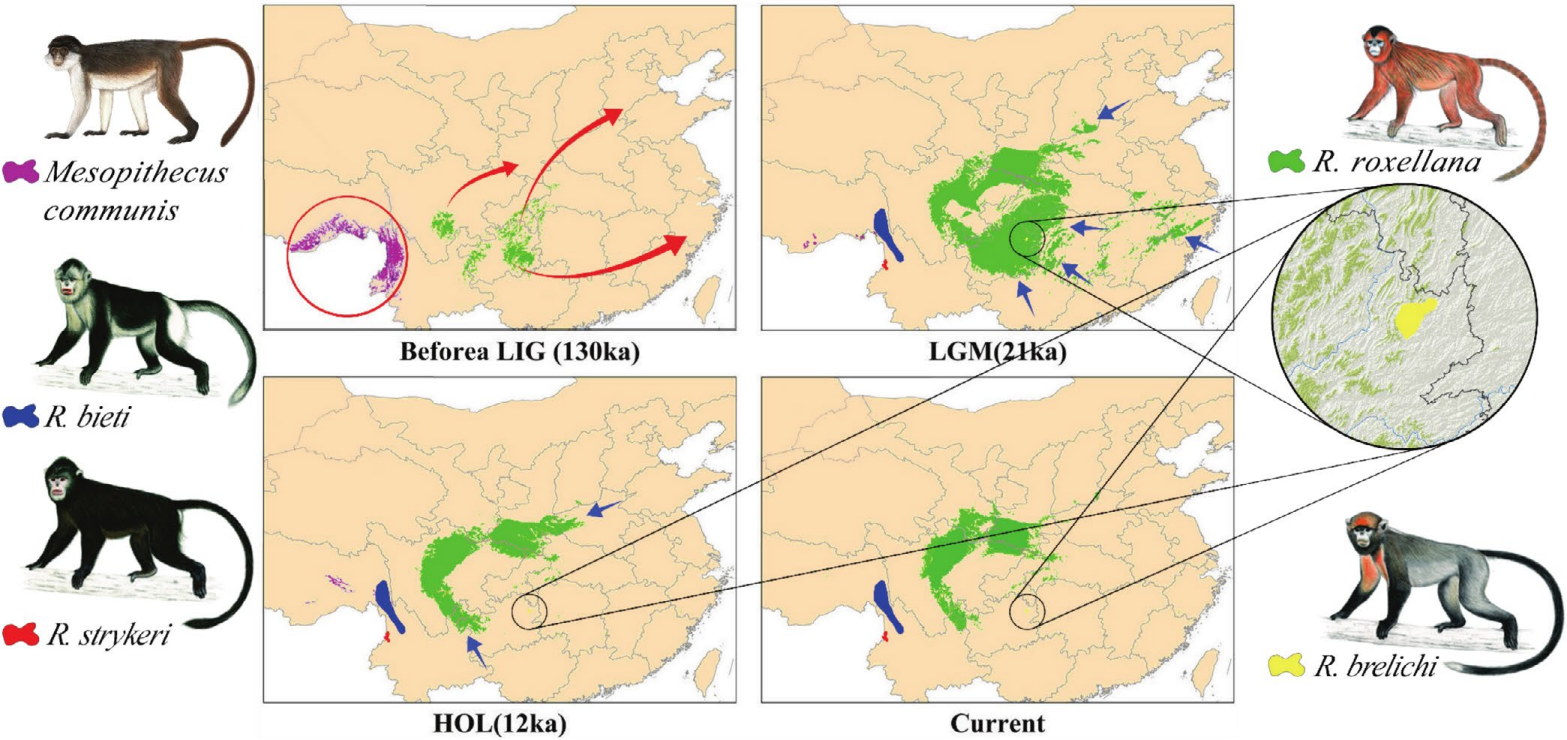
According to our recent publication demonstrating the relationship between ancestors and existing taxa of Asian colobines (Pan et al. 2025), and referring to the MaxEnt model, based on ecological niche, demonstrating the past and current distributions of *Rhinopithecus roxellana* (Li, Huang, et al. 2020), as well as fossil distribution patterns (Li, Huang, et al. 2020; Zhang et al. 2022), geographic distribution trajectories of the *Rhinopithecus* are illustrated. Hol, Holocene; LGM, the last glacial maximum; and LIG, the Last Interglacial period. The current distribution areas of *R. bieti*, *R. strykeri* , and *R. brelichi* are referred to (Chen et al. 2022; Long et al. 2020; Xiang et al. 2009). Red arrows indicate the expansion direction of *R. roxellana* , while the blue ones show the retraction of its populations.

Some statements have been provided in the Supplementary to discuss and clarify the classification and evolutionary relationships among the species in *Rhinopithecus*, especially regarding *R. roxellana* and *R. brelichi* . They are summarized in Figure 1. Fossil *Rhinopithecus* found up to now in China can be regarded as the ancestors of *R. roxellana* , which used to broadly spread in China, reaching Taiwan; and *R. brelichi* is one of the populations left in Fanjingshan National Nature Reserve, Guizhou, by the former most recently (Xiang et al. 2012), following the retreat of *R. roxellana* after the LGM into the Early Holocene (Li, Huang, et al. 2020).

### 2.2 Historical Distribution Division

Referring to major social upheavals that have significantly impacted China's society, environment, and ecology and shaped animal distribution, recent Chinese history has been divided into six periods (Zhang et al. 2022). They include:

1. Before 1800, the environment remained largely pristine due to limited early agricultural activity and human settlement. Thus, although our data records for *R. roxellana* date back to 1304 ce, we chose to begin with the period before 1800.
2. Between 1800 and 1849, marked by the emergence of heavy environmental damage following engagements with Western powers during the First Opium War (1839–1842).
3. Between 1850 and 1899, this period was characterized by significant environmental and societal devastation during the Second Opium War (1856–1860) and significant social chaos caused by the Taiping Rebellion (1850–1864).
4. From 1900 to 1949, China's population grew to 450 million, expanding into previously unoccupied regions, which resulted in considerable shrinkage of the natural environment and ecological damage. China was also involved in the Sino‐Japanese War, part of the Second World War, causing further environmental devastation.
5. Between 1950 and 1999, the period was marked by significant human population growth, exceeding one billion, as well as social and economic development, which led to increased demands for natural resources, including deforestation.
6. From 2000 to 2020, the real estate property market and the exploitation of natural resources for raw products were prominent features.

### 2.3 Field Surveys

The most recently surveyed demographic figure for the species was conducted in four provinces in China, where it dwells (Dai et al. 2024). Thus, between January 2018 and November 2019, we conducted a ground survey to determine the population size of *R. roxellana* . We tracked the monkey groups from 9:00 a.m. to 5:00 p.m. and calculated the average amount of time they spent staying and sleeping. Thus, this project involved a larger‐scale census survey of the monkeys, crossing their whole distribution areas in Gansu, Sichuan, Shaanxi, and Hubei provinces, including 27 nature reserves. Please refer to (He et al. 2023) for details of how to conduct field surveys and obtaining population sizes of the species.

An index of species density (individuals/km^2^) was used to represent the quantity of species distribution within a particular ecological area. It was ranked into three scales: (1): 1 or less; (2): 1–2; and (3): more than 2.

### 2.4 Historical Distribution Area

Regarding historical distribution, numerous older references have recorded the distribution of *R. roxellana* (such as “猴”, “狖”, “禺”, and “狨”) with precise county designations in different periods (Dai et al. 2024; Zhang et al. 2022). The county names were updated, referring to *Zhongguo Gudaishi Dituji* (Guo 1980). We mapped the distribution of *R. roxellana* across the counties and calculated their total areas with ArcGIS 10.7.

### 2.5 Fragment Index

**FIGURE 2:**
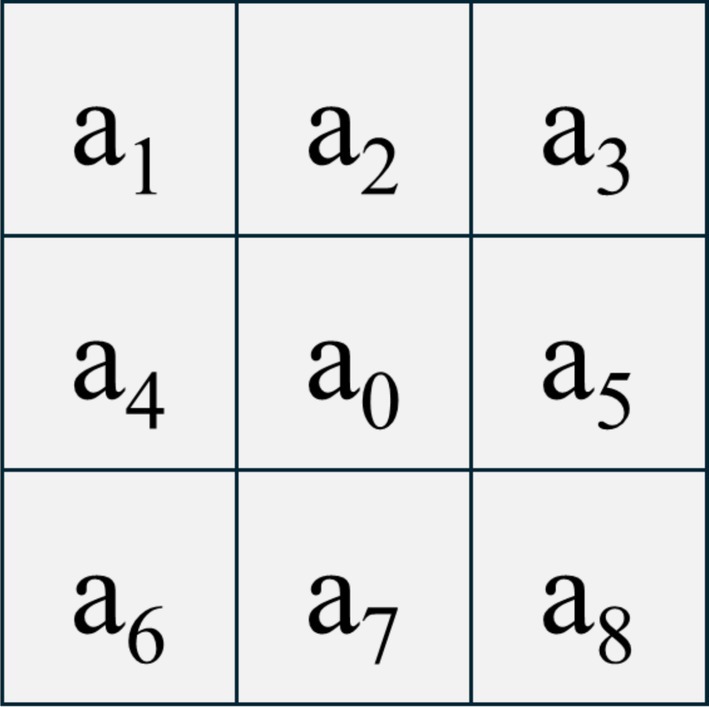
Calculation units designed in this study. a_0_: Where the monkeys were/are found, and a_1–8_: The neighboring grids of a_0_.

Referring to the Local Contrast Method (Chen et al. 2013), the fragment index of the *R. roxellana* is calculated by the following method. The distribution map was divided into individual grids with a 20 km × 20 km grid using ArcGIS. To calculate the fragmentation degree, we combine nine grids around it into a calculation unit (Figure 2). The FI of *R. roxellana* is represented by one minus the average of all calculation units.

(1) $$A_{\mathrm{jk}}=\frac{1}{9}\sum_{i=1}^{9}a_{\mathrm{ijk}}$$

(2) $${\mathrm{FI}}_{k}=1-\frac{1}{n}\sum_{j=1}^{n}A_{\mathrm{jk}}$$

Here, $a_{\mathrm{ijk}}$ is the *i*‐th grid in the *j*‐th calculation unit of the *k*‐th period; 1 indicates appearance within a grid, 0 means absence. $A_{\mathrm{jk}}$ is the value of the *j*‐th calculation unit in the *k*‐th period. ${\mathrm{FI}}_{k}$ is the fragmentation index of *R. roxellana* in the *k*‐th period.

## 3 Results

### 3.1 Fossil Distribution

**FIGURE 3:**
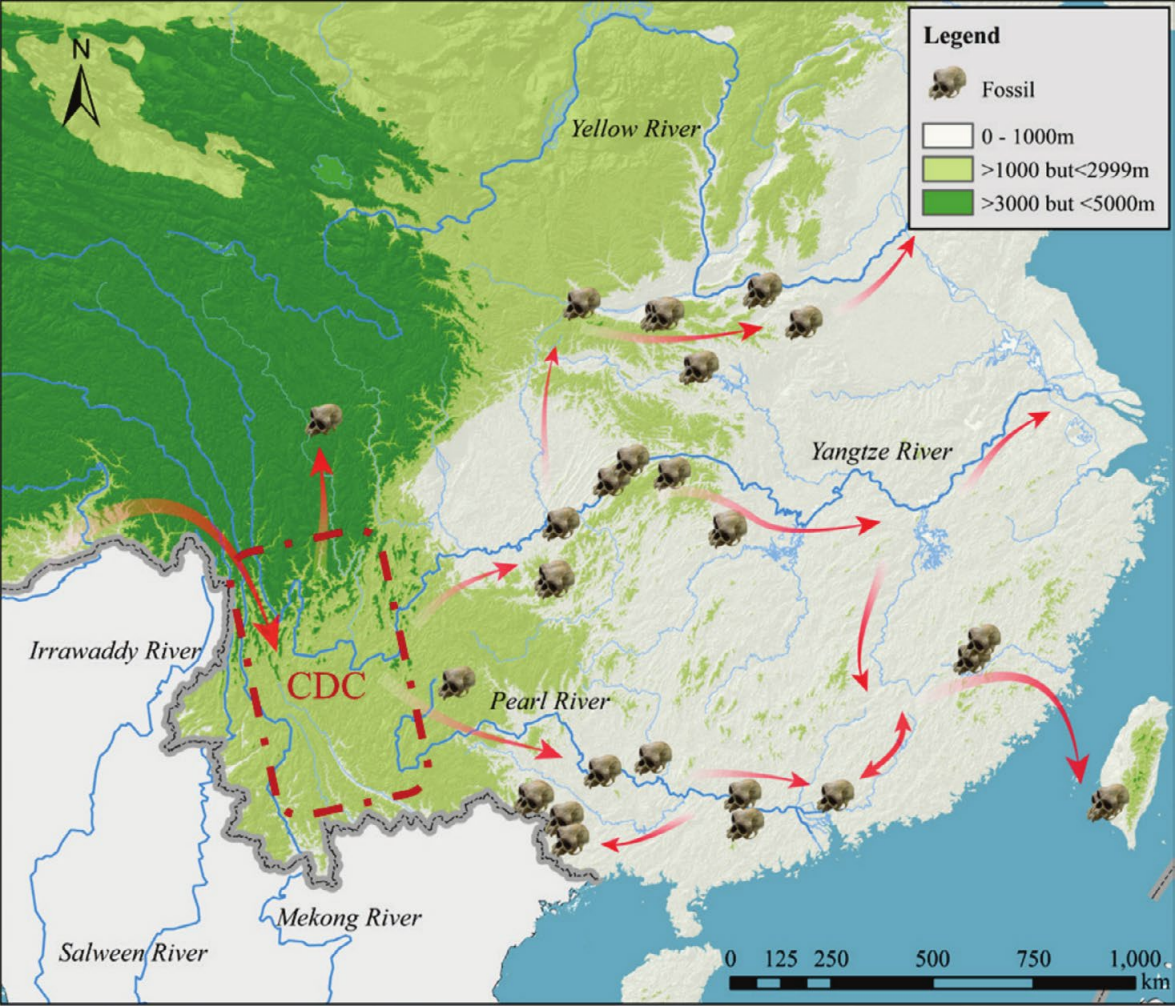
Dispersion and radiation of the ancestor of *R. roxellana—R. brelichi* on the mainland of Eastern Asia during the Pleistocene based on the fossil‐bearing sites. The Convergence‐Divergence Center (CDC) was referred to (Zhang et al. 2022).

Figure 3 illustrates the fossil dispersion and radiation of *R. roxellana* in mainland China and Taiwan during the Pleistocene, as well as the taxon's presence in Guizhou in the early Holocene (Figure 1 and Table S1). They originated from the Convergence‐Divergence Center, encompassing the Eastern Himalaya, the Southeastern Qinghai‐Tibet Plateau, and the surrounding mountains (Zhang et al. 2022). The monkeys migrated eastward along the Yangtze and Pearl Rivers but did not cross the Yellow River. The fact that fossils were found between the three major rivers implies that they radiated between and along waterways. The fossil found in Taiwan suggests that monkeys reached the island via land bridges formed during the Pleistocene and Early Holocene.

### 3.2 Historical Distribution During the Holocene

**FIGURE 4:**
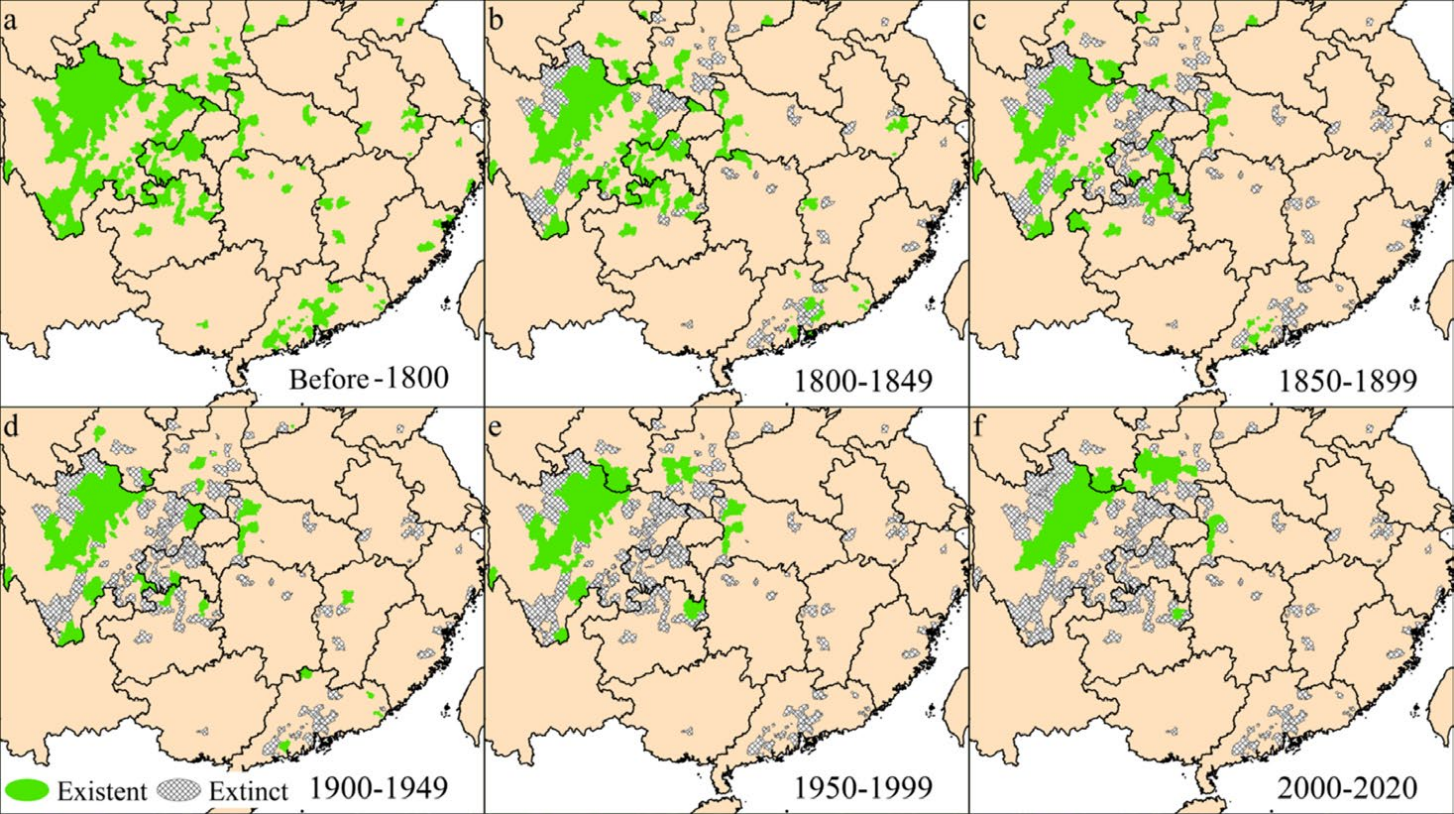
Historical distribution profile of *R. roxellana* and *R. brelichi* in China before 1800 (a); from 1800 to 1849 (b); from 1850 to 1899 (c); from 1900 to 1949 (d); from 1950 to 1999 (e); and from 2000 to 2020 (f).

According to the divisions, the historical distribution of *R. roxellana* , including *R. brelichi* , is illustrated in Figure 4 (Table S2). Prior to 1800 (a), the species were widely distributed in the southwestern region, primarily concentrated in Sichuan and its neighboring areas, as well as in the southern region, especially in Guangdong. Populations also spread in central, northern, and coastal regions, but with notable fragmentation. A significant disappearance occurred between 1800 and 1849 (b), primarily in Shaanxi, Hubei, Hunan, northeastern Yunnan, and the eastern coast of Guangdong. This phenomenon became more prominent during 1850–1899 (c), and remarkably reduced populations appeared in the Sichuan basin and adjacent areas. The period from 1900 to 1949 (d) saw further disappearances in Guangdong, Jiangxi, and the Sichuan Basin, forming a general pattern of current distribution, except for two isolated locations in Guangdong, one in Jiangxi, and another in Shanxi. The period from 1950 to 1999 (e) marks the extinction of isolated locations in Guangdong, Jiangxi, and Shanxi. The final period, from 2000 to 2020, shows an increasing population in Shaanxi and parts of Sichuan.

**FIGURE 5:**
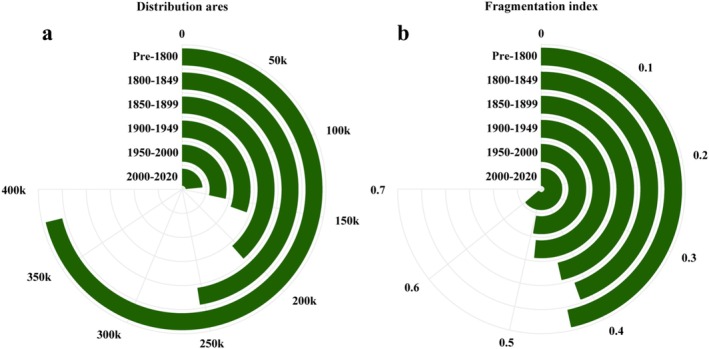
Historical changes in distribution areas (a) and fragmentation indices (b) of *R. roxellana* between pre‐1800 and 2020.

Periodic reductions in the distribution areas and changes in the fragmentation index are illustrated in Figure 5. Regarding the declining distribution sizes, notable shrinkages occurred during the periods 1800–1849, 1850–1899, and 1900–1949, three significant waves. The current distribution size has been generally established before the first part of the last century, after approximately 217,575 km^2^ had been lost between pre‐1800 and 1949 (Figure 5a). The corresponding fragmentation indices indicate that significant population isolation increased between 1900 and 1949 and between 2000 and 2020, with the value reaching its highest point (Figure 5b).

### 3.3 Current Demographic Profiles

The population records of *R. roxellana* in 27 nature reserves surveyed are listed in Table 1.

**TABLE 1:** The population size of *R. roxellana* and the nature reserve areas surveyed in this study.

Number	Province	Name	Population size	Area (km^2^)	Elevation
1	Shaanxi	Taibaishan	140	563.25	800–3771
2	Shaanxi	Huangbaiyuan	455	218.65	1280‐3120
3	Shaanxi	Niuweihe	235	150.04	800–3767
4	Shaanxi	Laoxiancheng	320	126.11	1524‐2838
5	Shaanxi	Zhouzhi	1150	563.93	1190‐2996
6	Shaanxi	Changqing	710	299.06	800–3071
7	Shaanxi	Foping	600	292.4	980–2904
8	Shaanxi	Guanyinshan	295	138.8	1150‐2574
9	Shaanxi	Tianhuashan	445	276.15	400–2893
10	Shaanxi	Huangguanshan	240	208.38	1200‐2893
11	Shaanxi	Pingheliang	105	154	1265‐2679
12	Shaanxi	Qinmuchuan	190	101.96	1000‐2054
13	Gansu	Yuhe	890	510.58	660–2472
14	Gansu	Baishuijiang	1275	2137.5	2200‐4800
15	Sichuan	Jiuzhaigou	170	643	2000‐4500
16	Sichuan	Wanglang	1100	322.97	2400‐4980
17	Sichuan	Tangjiahe	875	400	1150‐3864
18	Sichuan	Xuebaoding	575	636.15	1400‐5400
19	Sichuan	Xiaozhaizigou	560	443.85	1600‐4073
20	Sichuan	Qianfoshan	550	110.83	1630‐4047
21	Sichuan	Baishuihe	375	301.5	1480‐4818
22	Sichuan	Longxi‐Hongkou	630	310	1196‐4582
23	Sichuan	Siguniangshan	475	560	2750‐6250
24	Sichuan	Wolong	1300	2000	1100‐6250
25	Sichuan	Fengyongzhai	550	390.39	1000‐4896
26	Hubei	Shennongjia	1330	3253	1500‐3100
27	Hubei	Badang	440	209.09	240–3000

**FIGURE 6:**
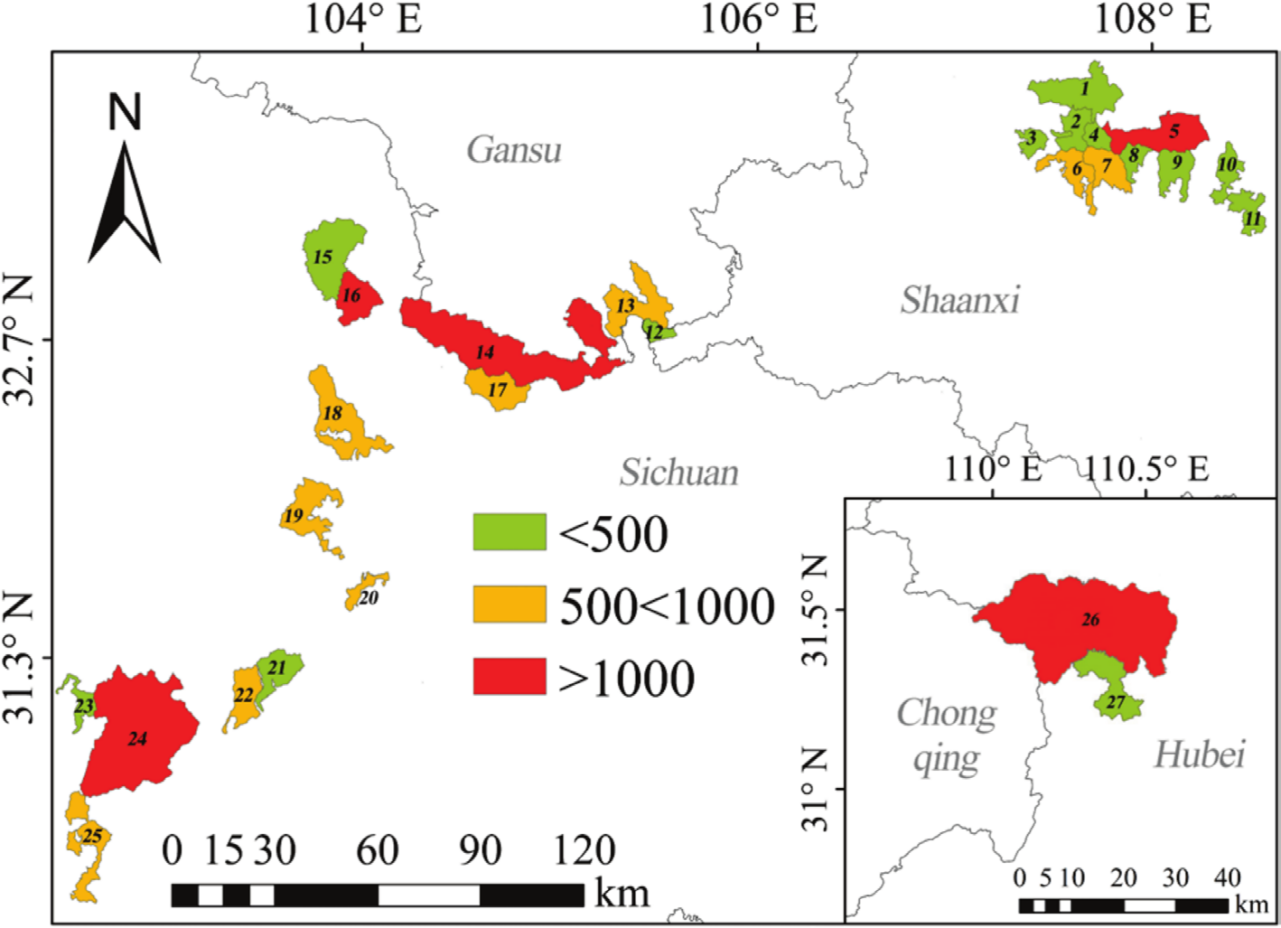
The population sizes of *R. roxellana* in the national nature reserves of China.

The variation in absolute individual numbers across different national nature reserves is presented in Figure 6. Those with population sizes between 500 and 1000 include six in Sichuan (17–20, 22, and 25), one in Gansu (13), and two in Shaanxi (6 and 7). Those less than 500 include three in Sichuan (15, 21, and 23), one in Gansu (12), and eight in Shaanxi (1–4, and 8–11).

**FIGURE 7:**
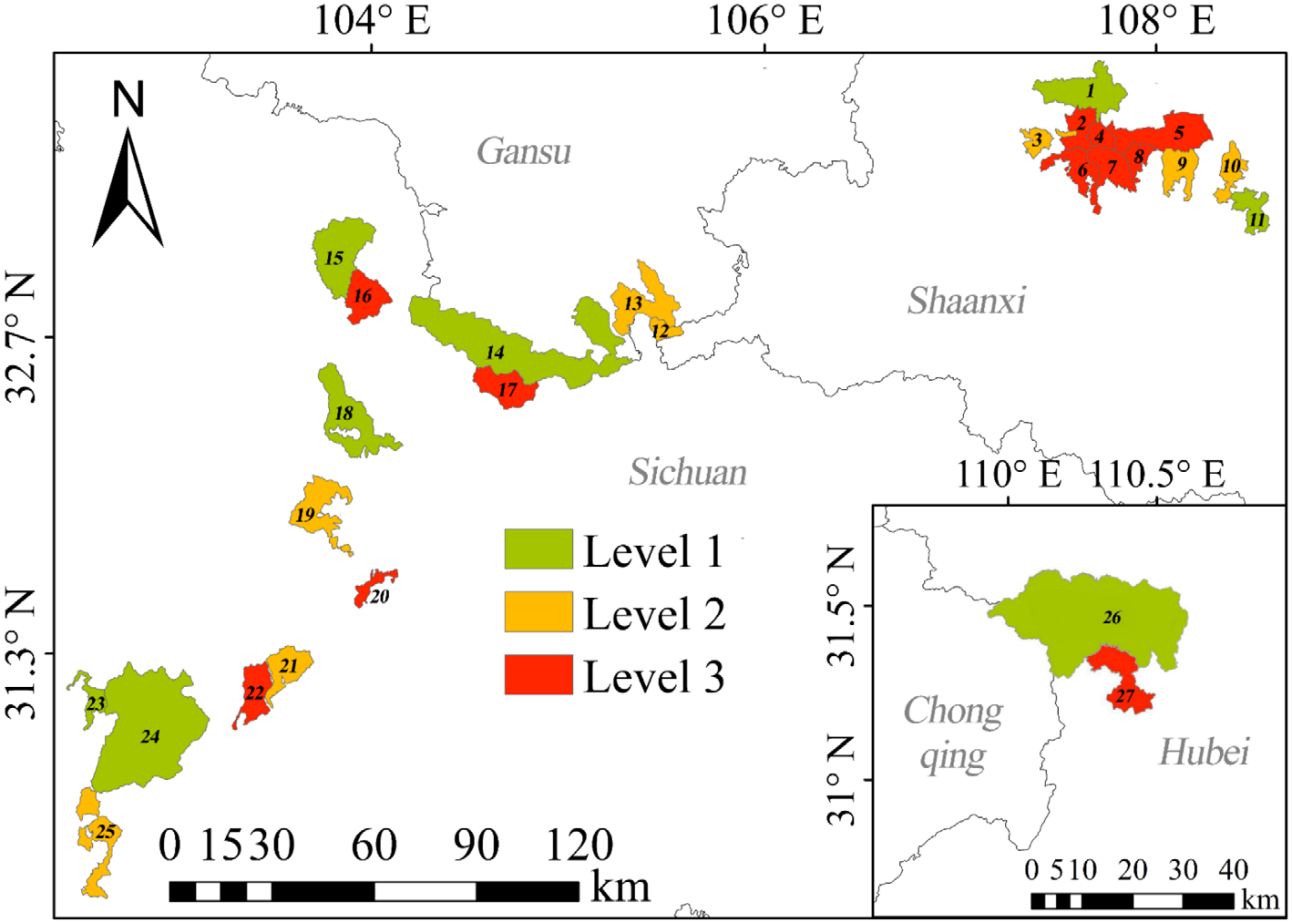
Regional density pattern of *R. roxellana* in the national nature reserve of China. Level 1: < 1/km^2^; level 2: 1–2/km^2^; and level 3: > 2/km^2^.

The species‐rich rate variation in the reserves is illustrated in Figure 7. Those with the highest rank (> 2/km^2^) include Shaanxi (2, 4, 5, 6, 7, and 8), Hubei (27), and Sichuan (16, 17, 20, and 22). Regarding the second level (1–2/km^2^), there are three in Shaanxi (3, 9, and 10), two in Gansu (12 and 13), and three in Sichuan (19, 21, and 25). Concerning the first level (< 1/km^2^), Hubei has one (26), Shaanxi has two (1 and 11), there is one in Gansu (14), and four in the latter (15, 18, 23, and 24).

An overall comparison of the population size, distribution area, and density of monkeys among nature reserves in four provinces is presented in Figure 7. Regarding population sizes, Sichuan has the largest, followed by Shaanxi, then Gansu, and finally, Hubei. Considering the distribution areas, Sichuan has the most critical proportion. Regarding monkey density, Sichuan has the highest density, followed by Shaanxi, Gansu, and Hubei, at level 1. Sichuan and Shaanxi show almost the same scale at level 2, which is higher than that of Gansu. Hubei is absent at this level. Sichuan and Shaanxi present a similar scale at the highest level (> 2/km^2^), which is higher than that of Hubei. However, Gansu lacks at this level.

## 4 Discussion

According to fossil‐bearing sites from the Pleistocene, *R. roxellana* is widely distributed across Eastern Asia (Figure 3). Some individuals eventually reached Taiwan through the land bridge between islands and the mainland, facilitated by lower sea levels during glaciation periods (Chang et al. 2012). It has been proposed that a colobine fossil from the Late Pliocene of Asia was found in central Japan, implying the ancestor of *Rhinopithecus* reached Japan (Maschenko 2003; Nishimura et al. 2012). The same scenarios occurred in other primates (macaques), resulting in the speciation of *Macaca cyclopis* in Taiwan, and *M. fuscata* in Japan (Li, He, et al. 2020).

As illustrated in Figure 4a, the distribution of *R. roxellana* before 1800 did not change significantly compared to its Pleistocene distribution pattern (Figure 3). They maintained the most extensive distribution area (Figure 5a). This confirms that during the Early Holocene, the environment and ecology in China were relatively primitive, regarded as a transitional period between ancient and modern Chinese society (Ho 1967; Marks 2011), in which the Chinese lived in small farming villages based on traditional agriculture. Southern and Southwestern China were home to the most extensive tropical and subtropical forests, which bore important fauna and flora (Ren et al. 2007).

Some populations of monkeys became fragmented along the central, northern, and eastern coasts, as well as in southern China. This is likely due to the origin and expansion of humans (*Homo erectus* and *H. sapiens* ) during the Late Pleistocene and Early Holocene, as shown in Figure 9 (Table S3). The fully anatomically modern human ( *H. sapiens* ) in southern China appeared in the Late Pleistocene (approximately 30,000–70,000 years ago) (Liu et al. 2015). Between 7500 and 1700 BP, they expanded further in China's coastal and southern regions following the Neolithic development, resulting in remarkable landscape transformations and the gradual occupation of the coastal plain. This facilitated the Neolithic transition from hunting and gathering to rice farming, alongside significant cultural development (Liu et al. 2021). Figure 4 illustrates the extraordinary decline of the species in the region after 1800, which is likely closely related to the significant human migration and expansion that occurred during this period.

This remarkable change was followed by the emergence of the earliest intricate societies in Northern China, specifically the Yangshao and Hongshan cultures along the Yellow River and the West Liao River civilization, which led to the development of significantly advanced agriculture (Ning et al. 2020). The increase in human activity (hunting and agriculture) resulted in the remarkable extinction of other animals (Huang et al. 2021; Li et al. 2002).

This significant disappearance intensified from 1850 to 1949 (Figure 3c,d), accompanied by a substantial reduction in distribution size (Figure 5a). However, there was no significant trend of increased fragmentation indices (Figure 5b), implying such a reduction was mainly caused by the shrunk distribution areas, as shown in Figure 3b,c. That change must be related to social and natural catastrophes in those areas, including the first Opium Wars in the Qing Dynasty between 1839 and 1842, which were primarily conducted in Guangdong, Fujian, Zhejiang, and Jiangsu, resulting in the reduction of many animal habitats in eastern and southern China between 1800 and 1849 (Hanes and Sanello 2005; Ouchterlony 2012). The Second Opium War (1856–1860) and the Taiping Rebellion (1850–1864) also increased social chaos, natural environmental and ecological damage, and overexploitation, which explicitly applied to the scenarios in Sichuan (Phillips 2011; Wang 1990), with a noticeable monkey population diminishing between 1850 and 1949 in its basin (Figure 3c,d). Thus, unlike the populations in the mountains, the marked population reduction of *R. roxellana* in the basin is closely tied to rapid human social development, where the human population increased dramatically—51.34 million in 1930 and 57.30 million in 1949, the most extraordinary population size for China at that time (Yue et al. 2005). That was promoted by its Baodun Culture (4600–3700 BP), the earliest archeological culture established in Southwestern China (Huang et al. 2019). Such rapid human social development has led to extensive population growth and environmental and habitat damage, resulting in significant impacts on local fauna and flora (Binford et al. 2017; Dong et al. 2017; Guedes et al. 2015; Zeng et al. 2016).

Further, during the Second World War (from 1939 to 1945), Sichuan was the stronghold of the Nationalist Party confronting the Japanese (Cheng 1999), with vigorously developed transportation, military industry, and agriculture, while suffering significant war devastation during this turbulent period, such as being heavily bombed (Li 2005).

Monkey populations in Guangdong and Jiangxi were extirpated during the 1950–1999 period (Li et al. 2002) (Figure 4e). During this period, China experienced unparalleled growth in many aspects, including agricultural expansion and rapid economic growth (Gabriel 1998), as well as substantial population growth (Jiang et al. 2018).

Environmental and ecological changes, as well as human‐induced activities, across the six periods also triggered large‐scale deforestation in China (Pan et al. 2016). That has been accelerated by increasing human encroachment into animals' habitats and a significant increase in human‐monkey encounters (Perkins 2017). The considerable increase in ecological fragmentation after 1949 (Figure 5b) echoes a profound impact on species distribution and survival.

**FIGURE 8:**
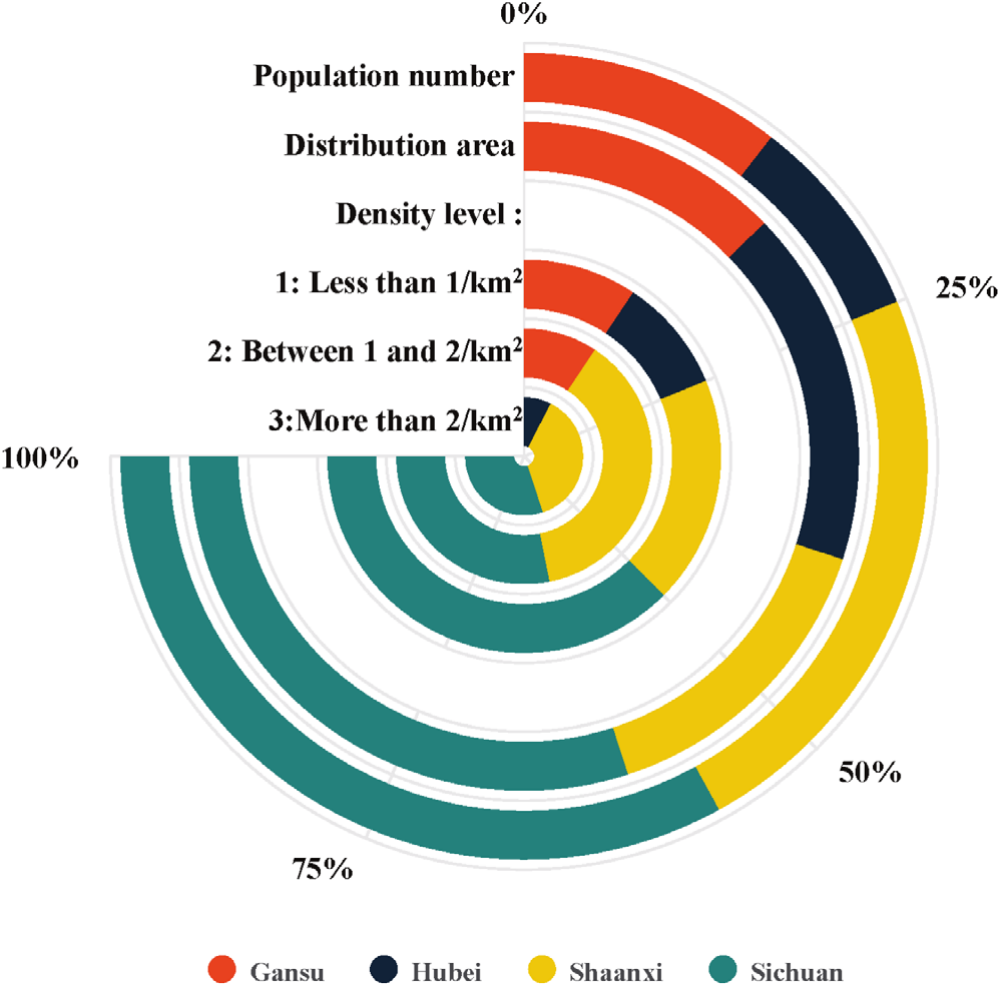
The comparison of population number, reserve area, and population density of *R. roxellana* in Gansu, Hubei, Shaanxi, and Sichuan.

Since the start of this century, a promising sign has been the reduced depletion of natural resources, and efforts to alleviate ecological impact have been conducted in some regions (Xiao et al. 2020; Zhao et al. 2021). Such efforts led to the regional expansion of suitable habitats and ecology for *R. roxellana* (Zhao et al. 2021). Over the decades, primary conservation efforts have focused explicitly on nature reserves and their surroundings, such as in Qinling, Shaanxi Province, as well as in Mongolia, Gansu, Ningxia, Qinghai, Xinjiang, Tibet, Yunnan, and Guizhou (Zhao et al. 2021). *R. roxellana* has benefited in Shaanxi Province, as evidenced by its growing population (Table 1; Figures 3e,f and 8). This is likely associated with the banning of the logging industry and the programs to return farmland to its natural state, initiated in 2000, as well as the implementation of ecological migration since 2012, in which some peasants have moved out of conservation regions (Wang et al. 2023).

**FIGURE 9:**
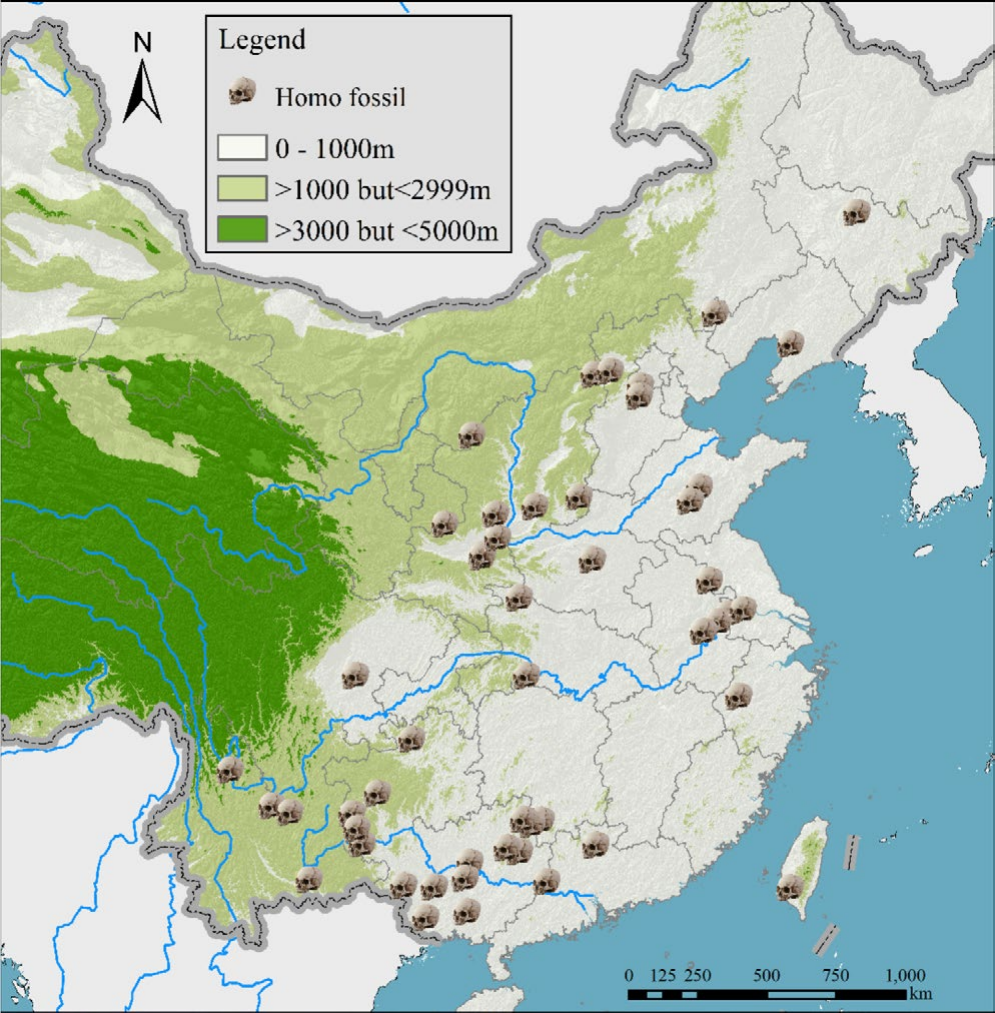
Fossil‐bearing sites of humans in China (*Homo erectus* and *H. sapiens* ) in the Pleistocene (Curnoe et al. 2012; Han et al. 2015; Li, Wang, et al. 2017; Li, Wu, et al. 2017; Liao et al. 2019; Liu 1999; Liu et al. 2002; Wang et al. 2011; Wu and Olsen 2009; Wu 2004; Wu and Poirier 1995).

As for other areas, however, anthropogenic pressures on the environment and ecology are still prominent. For example, land conversion for urbanization and the residential building industry increased remarkably from 17.92% in 1978 to 57.35% in 2016 (Zou et al. 2020). Consequently, this period features decreasing areas but increased ecological fragmentation (Figure 5a,b).

Thus, the results of this study indicate that the conservation status of *R. roxellana* varies among provinces and areas, such as in Qinling, where the population increased by approximately 31.0% during the 1980s, following the expansion of corridors between populations (Wang et al. 2023). Sichuan has a significant population size (50.0%) and a broader conservation area (Figures 5 and 6). A further conservation promotion in the region is to establish corridors between nature reserves, such as within Shennongjia. Wanglang Nature Reserve (Figures 5 and 6) should be the primary target for merging with Jiuzhaigou Nature Reserve, as it is the smallest area with the highest population density (Figure 6).

Another conservation challenge facing *R. roxellana* is the contradiction between conservation and local economic development, as well as the issue of unregulated eco‐tourism (Dai et al. 2024; Ma et al. 2024).

Thus, we propose the following measures to enhance the conservation status of the species.

1. Enlarging conservation areas. Currently, most of the reserves are surrounded by agricultural plantations and human activities, such as farming and grazing, creating a significant impact on the monkeys (Dai et al. 2024). The local villagers rely on firewood from the nature reserve due to the high costs of electricity and natural gas fees (DeWan et al. 2013). Thus, the government should assist locals with daily firewood consumption and encourage the use of fuel‐efficient stoves (DeWan et al. 2013; Xie and Shen 2021). Additionally, livestock farmers should be encouraged to plant fruit trees as a sustainable alternative that can reduce their economic dependence on natural resources.
2. Building animal corridors between adjacent conservation areas and enhancing supervision during the monkeys' breeding season, which mainly applies to the Nature Reservations of Xiaozhaizigou, Xuebaoding, Qianfoshan, and Fengyongzhai.
3. Regulating primate ecotourism. Unscientifically managed tours can negatively impact the monkeys' health, such as by causing psychological stress and increased aggression between individual monkeys (Yang et al. 2024; Zou et al. 2023).
4. Promoting effective public education on conservation and maintaining biodiversity is crucial in schools and society. The research and educational model presented here, which traces the monkeys' changes from the Pleistocene to the present and predicts their future growth, will offer a valuable tool in such an effort.

## 5 Conclusion

This study examines the evolutionary development of *Rhinopithecus roxellana* in China by analyzing fossil distribution from the Pleistocene, historical records during the Holocene, and the species' current geographic distribution. It investigates the impacts of geographic range contraction and human activities on the species. Additionally, the study provides a transect‐based review of population distribution disparities, offering scientific evidence and informing conservation strategies aimed at revising the species' current conservation status. The results indicate that:

1. Fossils of *Rhinopithecus* found up to now are primarily associated with *R. roxellana* ;
2. A significant range contraction of the species began in the early Holocene, coinciding with the expansion of modern human populations in central and coastal regions;
3. Although a notable population decline occurred in the early 20th century, closely linked to modern warfare, an unprecedented extinction trend emerged in the second half of the 20th century due to accelerating environmental degradation and habitat loss;
4. Sichuan is identified as the optimal province for the species' potential recovery and development. Another recent study analyzing with unmanned aerial vehicles (UAVs) indicates that the Giant Panda National Park in China's densely vegetated Qinling Mountains is also an ideal region for this species (He et al. 2023). Thus, they deserve to have a conservation priority without diminishing efforts in other areas.

## 6 Study Constraints

This project involved a comprehensive national survey encompassing 27 nature reserves across four provinces. Thus, some isolated monkey groups and individuals outside the reserves may have been overlooked in this survey. Therefore, the numbers in Table 1 may not reflect the actual repository of the species in China.

Similar to what occurs in East Asia with cercopithecines (Cercopithecinae), the fossil record of colobines is far from clear. Thus, fossils closely related to extant *Rhinopithecus* in southern China might also be linked to the Douc and Francois's (*Pygathrix* and *Trachypithecus*) that used to dwell in these regions (Takai et al. 2014).

## Author Contributions

**Hao Pan:** writing – original draft (lead). **Kang Huang:** investigation (equal), methodology (equal). **Jing Wang:** data curation (equal). **Chengrui Song:** investigation (equal). **Felix Guo:** investigation (equal). **Xiao Zhang:** methodology (equal). **Zhengfeng Hu:** methodology (equal). **He Zhang:** writing – review and editing (equal). **Kexin Ji:** investigation (equal). **Shujun He:** software (equal). **Songtao Guo:** investigation (equal). **Dayong Li:** data curation (equal). **Wenyong Tian:** investigation (equal). **Haitao Zhao:** data curation (equal). **Jianbing Yue:** investigation (equal). **Ruliang Pan:** writing – review and editing (lead). **Zhihua Zhou:** investigation (equal). **Gang He:** funding acquisition (lead), investigation (lead). **Baoguo Li:** funding acquisition (equal), project administration (equal), supervision (equal).

## Ethics Statement

The authors have nothing to report.

## Conflicts of Interest

The authors declare no conflicts of interest.

## Supporting information


**Data S1:** ece372036‐sup‐0001‐Supinfo.docx.

## Data Availability

All the required data are uploaded as Supporting Information—S1.
