# Supplementary material for: Evolutionary Distribution Changes of Sichuan Golden Monkeys ( Rhinopithecus roxellana ) in the Quaternary
Source: Ecol Evol. 2025 Sep 2;15(9):e72036. doi: 10.1002/ece3.72036 (PMC12405596; doi:10.1002/ece3.72036)
Supplement: Supplementary file 1 — Data S1: ece372036‐sup‐0001‐Supinfo.docx. [file ECE3-15-e72036-s001.docx]

Supplementary

Evolutionary and classification summary of *Rhinopithecus*

Scholars have studied fossil locations and classifications related to *Rhinopithecus* in China (Gu & Hu, 1991; Jablonski, 2008; Jablonski & Gu, 1991; Li et al., 2002; Zhang et al., 2022). However, no study has specifically delineated the fossil distribution boundaries of the four species (*R. bieti, R. strykeri, R. brelichi*, and *R. roxellana*). Thus, the first step of this study is to clarify the fossil distribution boundary closely associated with the species.

Among the four species, *R. strykeri* and *R. bieti* geographically differ from *R. roxellana* and *R. brelichi* (Figure 1). The former two are considered part of the Himalayan group. In contrast, the latter two are classified as part of the northern group (Kuang et al., 2023). *R. strykeri* is confined to very narrow areas in Mt. Gaoligong, and *R. bieti* in upper streams between the Mekong and Yangtze Rivers (Li et al., 2024; Pan et al., 2024a; Xiang et al., 2007). The fossils regarded as colobines found in the same area are only those of *Mesopithecus pentelicus,* unearthed in Shuitangba, Zhaotong, Yunnan Province (Ji et al., 2020; Pan et al., 2025). They, however, are considered to be associated with langurs and odd-nosed colobines in the region (Jablonski et al., 2020; Pan et al., 2024b). In other words, no fossils have been identified as direct relatives of the two extant *Rhinopithecus* species in these areas. Thus, *Rhinopithecus* fossils unearthed in China and published are generally related to the taxa of *R.* *roxellana* and *R. brelichi* (Li et al., 2002; Li et al., 2024). These fossils, excavated in Sichuan, Shaanxi, Henan, and Hubei, are considered ancestors of *R. roxellana* in the Early to Middle Pleistocene (Chang et al., 2012; Colbert & Hooijer, 1953; Gu & Hu, 1991; Gu & Jablonski, 1989; Jablonski, 1998; Pan & Jablonski, 1987). They are located in the regions of the Northern bank of the Yangtze River.

**Figure 1 about here**

Over the years, scholars have debated the *Rhinopithecus* fossils found in the Zhejiang, Fujian, Guangdong, Guangxi, Hunan, and Guizhou regions, which encompass the southern regions of the Yangtze River and Pearl River areas. Thus, geographically, they could be regarded as the ancestors of *R. roxellana* and/or *R. brelichi.*

Recent studies have provided evidence to clarify this debate.

1) Genetic evidence. Among the three taxa of *Rhinopithecus*, the ancestor of *R. bieti* could occur at ~ 1.33 mya, while those for *R. roxellana* and *R. brelichi* are estimated at ~0.91 mya and ~0.36 mya, respectively (Yang et al., 2012). *R. brelichi* is the youngest of the three, and it has been considered an extended taxon of *R. roxellana*, found in Fanjingshan, Guizhou. Therefore, the two species can be regarded as a monophyletic clade and separated most recently (Liedigk et al., 2012). The same study also proposes that speciation within *Rhinopithecus* in China may have occurred in the Early Pleistocene (approximately 2.6 million years ago). The separation between *R. roxellana and R. brelichi* is approximately 1.69 mya – the genetic distance between them is shorter than that of any other pair within the genus. Genetically, *R. brelichi* has only a single haplotype, the lowest compared with the other species in *Rhinopithecus* in China.

2) The geographic distribution. Like other animals, glaciation during the Quaternary significantly shaped the current distribution patterns of *R. roxellana* and *R. brelichi*. According to the MaxEnt model of probability distribution, *R. roxellana* expanded into the region where *R. brelichi* is found during the Last Glacial Maximum (LGM, 21 ka) in Guizhou (Figure 1). However, this range began shrinking during the Early Holocene (12 ka), leaving *R. brelichi* isolated from *R. roxellana* (Li et al., 2020).

3) The physical structures of the Yangtze and Pearl Rivers were formed before the Pliocene; primates in Eastern Asia completed their dispersion and radiation along them during the Pleistocene (Zhang et al., 2022). The rivers frequently changed direction during the glaciation. Such alterations likely provided channels and paths for animals to migrate between Southern and Northern China by crossing rivers during the Pleistocene and Early Holocene, allowing many animals, such as the rhesus monkey (*Macaca mulatta*), to spread broadly across Northern, Southern, and Eastern China. The Tibetan macaque (*M. thibetana*) crossed the Yangtze and Pearl Rivers, reaching Guangxi and Guangdong (Li et al., 2024). Furthermore, the distribution of tigers (*Panthera tigris*) was extensive in China, spanning across the Yangtze and Pearl Rivers during the period (Kang et al., 2010). Thus, it is possible that *R. roxellana* crossed the Yangtze and Pearl Rivers, reaching Guangxi and Guangdong in southern China, and ultimately Taiwan via a land bridge; however, it became extinct there in the Late Pleistocene (Chang et al., 2012).

Table S1. The recorded fossil sites of *Rhinopithecus roxellana* used in this study, based on which Figure 3 was generated.

Table S1. Location of *Rhinopithecus roxellana* fossil sites in China

| **Number** | **Nation** | **Group** | **Family** | **Subfamily** | **Genus** | **Species** | **Province** | **Location** |
| --- | --- | --- | --- | --- | --- | --- | --- | --- |
| 1 | China | Haplorhines | Cercopithecidae | Colobinae | *Rhinopithecus* | *sp.* | Chongqing | Chongqing |
| 2 | China | Haplorhines | Cercopithecidae | Colobinae | *Rhinopithecus* | *sp.* | Fujian | Mingxi |
| 3 | China | Haplorhines | Cercopithecidae | Colobinae | *Rhinopithecus* | *sp.* | Fujian | Jiangle |
| 4 | China | Haplorhines | Cercopithecidae | Colobinae | *Rhinopithecus* | *sp.* | Guangdong | Fengkai |
| 5 | China | Haplorhines | Cercopithecidae | Colobinae | *Rhinopithecus* | *sp.* | Guangdong | Luoding |
| 6 | China | Haplorhines | Cercopithecidae | Colobinae | *Rhinopithecus* | *sp.* | Guangdong | Guangdong |
| 7 | China | Haplorhines | Cercopithecidae | Colobinae | *Rhinopithecus* | *sp.* | Guangxi | Daxin |
| 8 | China | Haplorhines | Cercopithecidae | Colobinae | *Rhinopithecus* | *sp.* | Guangxi | Duan |
| 9 | China | Haplorhines | Cercopithecidae | Colobinae | *Rhinopithecus* | *sp.* | Guangxi | Liujiang |
| 10 | China | Haplorhines | Cercopithecidae | Colobinae | *Rhinopithecus* | *sp.* | Guizhou | Pan Xian |
| 11 | China | Haplorhines | Cercopithecidae | Colobinae | *Rhinopithecus* | *sp.* | Guizhou | Pan Xian |
| 12 | China | Haplorhines | Cercopithecidae | Colobinae | *Rhinopithecus* | *sp.* | Guizhou | Tongzi |
| 13 | China | Haplorhines | Cercopithecidae | Colobinae | *Rhinopithecus* | *sp.* | Henan | Xinan |
| 14 | China | Haplorhines | Cercopithecidae | Colobinae | *Rhinopithecus* | *sp.* | Hubei | Jianshi |
| 15 | China | Haplorhines | Cercopithecidae | Colobinae | *Rhinopithecus* | *sp.* | Hubei | Yunxi |
| 16 | China | Haplorhines | Cercopithecidae | Colobinae | *Rhinopithecus* | *sp.* | Hubei | Cili |
| 17 | China | Haplorhines | Cercopithecidae | Colobinae | *Rhinopithecus* | *sp.* | Hubei | Yun |
| 18 | China | Haplorhines | Cercopithecidae | Colobinae | *Rhinopithecus* | *sp.* | Shaanxi | Baoji |
| 19 | China | Haplorhines | Cercopithecidae | Colobinae | *Rhinopithecus* | *sp.* | Shaanxi | Lantian |
| 20 | China | Haplorhines | Cercopithecidae | Colobinae | *Rhinopithecus* | *sp.* | Sichuan | Luhuo |
| 21 | China | Haplorhines | Cercopithecidae | Colobinae | *Rhinopithecus* | *lantianensis* | Hubei | Xuetangliangzi |
| 22 | China | Haplorhines | Cercopithecidae | Colobinae | *Rhinopithecus* | *lantianensis* | Shaanxi | Lantian |
| 23 | China | Haplorhines | Cercopithecidae | Colobinae | *Rhinopithecus* | *lantianensis* | Shaanxi | Gongwangling |
| 24 | China | Haplorhines | Cercopithecidae | Colobinae | *Rhinopithecus* | *lantianensis* | Sichuan | Wanxian |
| 25 | China | Haplorhines | Cercopithecidae | Colobinae | *Rhinopithecus* | *roxellana* | Henan | Xinan |
| 26 | China | Haplorhines | Cercopithecidae | Colobinae | *Rhinopithecus* | *sp.* | Fujan | Cili |
| 27 | China | Haplorhines | Cercopithecidae | Colobinae | *Rhinopithecus* | *sp.* | Guangxi | Du’an |
| 28 | China | Haplorhines | Cercopithecidae | Colobinae | *Rhinopithecus* | *sp.* | Guangxi | Chongzuo |
| 29 | China | Haplorhines | Cercopithecidae | Colobinae | *Rhinopithecus* | *sp.* | Taiwan | Chochen |
| 30 | China | Haplorhines | Cercopithecidae | Colobinae | *Rhinopithecus* | *sp.* | Taiwan | Chochen |
| 31 | China | Haplorhines | Cercopithecidae | Colobinae | *Rhinopithecus* | *tingianus* | Guangxi | Du’an |
| 32 | China | Haplorhines | Cercopithecidae | Colobinae | *Rhinopithecus* | *tingianus* | Guangxi | Niushuishan |
| 33 | China | Haplorhines | Cercopithecidae | Colobinae | *Rhinopithecus* | *tingianus* | Guizhou | Tongzi |
| 34 | China | Haplorhines | Cercopithecidae | Colobinae | *Rhinopithecus* | *tingianus* | Hunan | Cili |
| 35 | China | Haplorhines | Cercopithecidae | Colobinae | *Rhinopithecus* | *tingianus* | Sichuan | Yenchingkuo |

Table S2. The historical distribution sites of *Rhinopithecus roxellana* from 183 AD to 2020 AD in China, based on which Figure 4 was generated.

Table S2. Historical distribution sites of *Rhinopithecus roxellana* in China.

| **Number** | **Genus** | **Species** | **Provinces** | **Location** | **longitude** | **Latitude** | **year** |
| --- | --- | --- | --- | --- | --- | --- | --- |
| 1 | *Rhinopithecus* | *sp.* | Guangdong | Dongguan | 113.76 | 23.03 | 1304 |
| 2 | *Rhinopithecus* | *sp.* | Guangdong | Forshan | 113.13 | 23.03 | 1304 |
| 3 | *Rhinopithecus* | *sp.* | Guangdong | Guangzhou | 113.27 | 23.14 | 1304 |
| 4 | *Rhinopithecus* | *sp.* | Guangdong | Jiangmen | 113.09 | 22.58 | 1304 |
| 5 | *Rhinopithecus* | *sp.* | Guangdong | Qingyuan | 113.06 | 23.69 | 1304 |
| 6 | *Rhinopithecus* | *sp.* | Guangdong | Shenzhen | 114.06 | 22.55 | 1304 |
| 7 | *Rhinopithecus* | *sp.* | Guangdong | Zhongshan | 113.4 | 22.52 | 1304 |
| 8 | *Rhinopithecus* | *sp.* | Guangdong | Zhuhai | 113.58 | 22.28 | 1304 |
| 9 | *Rhinopithecus* | *sp.* | Zhejiang | Leqin | 120.99 | 28.12 | 1413 |
| 10 | *Rhinopithecus* | *sp.* | Hunan | Changde | 111.71 | 29.04 | 1496 |
| 11 | *Rhinopithecus* | *sp.* | Hunan | Yiyang | 112.36 | 28.56 | 1496 |
| 12 | *Rhinopithecus* | *sp.* | Hunan | Yueyang | 113.14 | 29.36 | 1496 |
| 13 | *Rhinopithecus* | *sp.* | Hunan | Zhangjiajie | 110.49 | 29.12 | 1496 |
| 14 | *Rhinopithecus* | *sp.* | Jiangxi | Pingxiang | 113.86 | 27.63 | 1513 |
| 15 | *Rhinopithecus* | *sp.* | Jiangxi | Xinyu | 114.92 | 27.82 | 1513 |
| 16 | *Rhinopithecus* | *sp.* | Jiangxi | Yichun | 114.42 | 27.82 | 1513 |
| 17 | *Rhinopithecus* | *sp.* | Anhui | Dangtu | 118.5 | 31.58 | 1531 |
| 18 | *Rhinopithecus* | *sp.* | Anhui | Wuhu | 118.44 | 31.36 | 1531 |
| 19 | *Rhinopithecus* | *sp.* | Fujian | Ningde | 119.55 | 26.67 | 1544 |
| 20 | *Rhinopithecus* | *sp.* | Fujian | Xiapu | 120.01 | 26.89 | 1544 |
| 21 | *Rhinopithecus* | *sp.* | Guangdong | Zengcheng | 113.82 | 23.27 | 1544 |
| 22 | *Rhinopithecus* | *sp.* | Jiangxi | Pingxiang | 113.86 | 27.63 | 1544 |
| 23 | *Rhinopithecus* | *sp.* | Jiangxi | Xinyu | 114.92 | 27.82 | 1544 |
| 24 | *Rhinopithecus* | *sp.* | Jiangxi | Yichun | 114.42 | 27.82 | 1544 |
| 25 | *Rhinopithecus* | *sp.* | Shaanxi | Luoyang | 106.16 | 33.33 | 1544 |
| 26 | *Rhinopithecus* | *sp.* | Shaanxi | Shangluo | 109.92 | 33.88 | 1547 |
| 27 | *Rhinopithecus* | *sp.* | Shaanxi | Tongchuan | 109.08 | 35.07 | 1547 |
| 28 | *Rhinopithecus* | *sp.* | Shaanxi | Weinan | 109.52 | 34.51 | 1547 |
| 29 | *Rhinopithecus* | *sp.* | Shaanxi | Xianyang | 108.72 | 34.34 | 1547 |
| 30 | *Rhinopithecus* | *sp.* | Hunan | Changde | 111.71 | 29.04 | 1569 |
| 31 | *Rhinopithecus* | *sp.* | Hunan | Yiyang | 112.36 | 28.56 | 1569 |
| 32 | *Rhinopithecus* | *sp.* | Hunan | Yueyang | 113.14 | 29.36 | 1569 |
| 33 | *Rhinopithecus* | *sp.* | Hunan | Zhangjiajie | 110.49 | 29.12 | 1569 |
| 34 | *Rhinopithecus* | *roxellana* | shaanxi | Baoji | 107.24 | 34.36 | 1664 |
| 35 | *Rhinopithecus* | *sp.* | shaanxi | Baoji | 107.24 | 34.36 | 1664 |
| 36 | *Rhinopithecus* | *sp.* | Shaanxi | Tongchuan | 109.08 | 35.07 | 1667 |
| 37 | *Rhinopithecus* | *sp.* | Shaanxi | Weinan | 109.52 | 34.51 | 1667 |
| 38 | *Rhinopithecus* | *sp.* | Shaanxi | Xianyang | 108.72 | 34.34 | 1667 |
| 39 | *Rhinopithecus* | *sp.* | Shaanxi | ZHashui | 109.12 | 33.69 | 1667 |
| 40 | *Rhinopithecus* | *sp.* | Hubei | Anlu | 113.7 | 31.26 | 1669 |
| 41 | *Rhinopithecus* | *sp.* | Hubei | Suizhou | 113.39 | 31.7 | 1669 |
| 42 | *Rhinopithecus* | *sp.* | Anhui | Dangtu | 118.5 | 31.58 | 1673 |
| 43 | *Rhinopithecus* | *sp.* | Anhui | Wuhu | 118.44 | 31.36 | 1673 |
| 44 | *Rhinopithecus* | *sp.* | Guangdong | Yangchun | 111.8 | 22.18 | 1687 |
| 45 | *Rhinopithecus* | *sp.* | Guangdong | Forshan | 113.13 | 23.03 | 1691 |
| 46 | *Rhinopithecus* | *roxellana* | fujian | Yongtai | 118.93 | 25.87 | 1701 |
| 47 | *Rhinopithecus* | *roxellana* | guangdong | Conghua | 108.49 | 27.92 | 1701 |
| 48 | *Rhinopithecus* | *roxellana* | jiangxi | Yichun | 114.41 | 27.81 | 1701 |
| 49 | *Rhinopithecus* | *sp.* | fujian | Yongtai | 118.93 | 25.87 | 1701 |
| 50 | *Rhinopithecus* | *sp.* | guangdong | Conghua | 108.49 | 27.92 | 1701 |
| 51 | *Rhinopithecus* | *sp.* | jiangxi | Yichun | 114.41 | 27.81 | 1701 |
| 52 | *Rhinopithecus* | *sp.* | Guangdong | Conghua | 113.59 | 23.55 | 1710 |
| 53 | *Rhinopithecus* | *roxellana* | shaanxi | Longxian | 106.86 | 34.89 | 1713 |
| 54 | *Rhinopithecus* | *sp.* | Shaanxi | Longxian | 106.87 | 34.9 | 1713 |
| 55 | *Rhinopithecus* | *sp.* | shaanxi | Longxian | 106.86 | 34.89 | 1713 |
| 56 | *Rhinopithecus* | *sp.* | Guangdong | Conghua | 113.59 | 23.55 | 1730 |
| 57 | *Rhinopithecus* | *sp.* | Guangdong | Lianpin | 114.5 | 24.38 | 1730 |
| 58 | *Rhinopithecus* | *sp.* | Shaanxi | Xunyang | 109.37 | 32.84 | 1731 |
| 59 | *Rhinopithecus* | *sp.* | Shandong | Linyi | 118.36 | 35.11 | 1736 |
| 60 | *Rhinopithecus* | *sp.* | Zhejiang | Shaoxing | 120.59 | 30.04 | 1736 |
| 61 | *Rhinopithecus* | *roxellana* | sichuan | Baoxing | 102.81 | 30.37 | 1739 |
| 62 | *Rhinopithecus* | *roxellana* | sichuan | Ganzi | 101.96 | 30.05 | 1739 |
| 63 | *Rhinopithecus* | *roxellana* | sichuan | Hanyuan | 102.65 | 29.34 | 1739 |
| 64 | *Rhinopithecus* | *roxellana* | sichuan | Lushan | 104.56 | 30.52 | 1739 |
| 65 | *Rhinopithecus* | *roxellana* | sichuan | Mingshan | 103.11 | 30.07 | 1739 |
| 66 | *Rhinopithecus* | *roxellana* | sichuan | Neijiang | 105.05 | 29.58 | 1739 |
| 67 | *Rhinopithecus* | *roxellana* | sichuan | QInxi | 103.92 | 29.15 | 1739 |
| 68 | *Rhinopithecus* | *roxellana* | sichuan | Tianquan | 102.77 | 30.61 | 1739 |
| 69 | *Rhinopithecus* | *roxellana* | sichuan | Xiaojin | 102.36 | 31.01 | 1739 |
| 70 | *Rhinopithecus* | *roxellana* | sichuan | Ya'an | 103.04 | 30.01 | 1739 |
| 71 | *Rhinopithecus* | *sp.* | sichuan | Baoxing | 102.81 | 30.37 | 1739 |
| 72 | *Rhinopithecus* | *sp.* | sichuan | Ganzi | 101.96 | 30.05 | 1739 |
| 73 | *Rhinopithecus* | *sp.* | sichuan | Hanyuan | 102.65 | 29.34 | 1739 |
| 74 | *Rhinopithecus* | *sp.* | sichuan | Lushan | 104.56 | 30.52 | 1739 |
| 75 | *Rhinopithecus* | *sp.* | sichuan | Mingshan | 103.11 | 30.07 | 1739 |
| 76 | *Rhinopithecus* | *sp.* | sichuan | Neijiang | 105.05 | 29.58 | 1739 |
| 77 | *Rhinopithecus* | *sp.* | sichuan | QInxi | 103.92 | 29.15 | 1739 |
| 78 | *Rhinopithecus* | *sp.* | sichuan | Tianquan | 102.77 | 30.61 | 1739 |
| 79 | *Rhinopithecus* | *sp.* | sichuan | Xiaojin | 102.36 | 31.01 | 1739 |
| 80 | *Rhinopithecus* | *sp.* | sichuan | Ya'an | 103.04 | 30.01 | 1739 |
| 81 | *Rhinopithecus* | *sp.* | Sichuan | Ya'an | 103.05 | 30.02 | 1739 |
| 82 | *Rhinopithecus* | *sp.* | Anhui | Dangtu | 118.5 | 31.58 | 1757 |
| 83 | *Rhinopithecus* | *sp.* | Anhui | Wuhu | 118.44 | 31.36 | 1757 |
| 84 | *Rhinopithecus* | *sp.* | Guangdong | Boluo | 114.3 | 23.18 | 1763 |
| 85 | *Rhinopithecus* | *sp.* | Hubei | Yichang | 111.29 | 30.7 | 1763 |
| 86 | *Rhinopithecus* | *roxellana* | Gansu | Qingling | 105.72 | 34.58 | 1764 |
| 87 | *Rhinopithecus* | *roxellana* | sichuan | Nanchong | 106.11 | 30.84 | 1764 |
| 88 | *Rhinopithecus* | *sp.* | Gansu | Chenxian | 105.75 | 33.76 | 1764 |
| 89 | *Rhinopithecus* | *sp.* | Gansu | Lixina | 105.18 | 34.19 | 1764 |
| 90 | *Rhinopithecus* | *sp.* | Gansu | Qingling | 105.72 | 34.58 | 1764 |
| 91 | *Rhinopithecus* | *sp.* | Gansu | Tianshui | 105.73 | 34.59 | 1764 |
| 92 | *Rhinopithecus* | *sp.* | Gansu | Xihe | 105.31 | 34.02 | 1764 |
| 93 | *Rhinopithecus* | *sp.* | sichuan | Nanchong | 106.11 | 30.84 | 1764 |
| 94 | *Rhinopithecus* | *sp.* | Shaanxi | Fengxiang | 107.41 | 34.53 | 1766 |
| 95 | *Rhinopithecus* | *sp.* | Shaanxi | Longxian | 106.87 | 34.9 | 1766 |
| 96 | *Rhinopithecus* | *sp.* | Gansu | Gangu | 105.34 | 34.74 | 1770 |
| 97 | *Rhinopithecus* | *sp.* | Shanxi | Linyi | 110.78 | 35.15 | 1773 |
| 98 | *Rhinopithecus* | *sp.* | Guangdong | Chaozhou | 116.63 | 23.66 | 1775 |
| 99 | *Rhinopithecus* | *sp.* | Guangdong | Jieyang | 116.38 | 23.56 | 1775 |
| 100 | *Rhinopithecus* | *sp.* | Guangdong | Meizhou | 116.13 | 24.29 | 1775 |
| 101 | *Rhinopithecus* | *sp.* | Guangdong | Shantou | 116.69 | 23.36 | 1775 |
| 102 | *Rhinopithecus* | *sp.* | Shaanxi | Ankang | 109.04 | 32.69 | 1779 |
| 103 | *Rhinopithecus* | *sp.* | Shaanxi | Shangluo | 109.92 | 33.88 | 1779 |
| 104 | *Rhinopithecus* | *sp.* | Shaanxi | Tongchuan | 109.08 | 35.07 | 1779 |
| 105 | *Rhinopithecus* | *sp.* | Shaanxi | Weinan | 109.52 | 34.51 | 1779 |
| 106 | *Rhinopithecus* | *sp.* | Shaanxi | XI'an | 108.95 | 34.35 | 1779 |
| 107 | *Rhinopithecus* | *sp.* | Shaanxi | Xianyang | 108.72 | 34.34 | 1779 |
| 108 | *Rhinopithecus* | *sp.* | Guizhou | Tongren | 109.2 | 27.74 | 1780 |
| 109 | *Rhinopithecus* | *roxellana* | sichuan | Guangyuan | 105.84 | 32.44 | 1785 |
| 110 | *Rhinopithecus* | *sp.* | sichuan | Guangyuan | 105.84 | 32.44 | 1785 |
| 111 | *Rhinopithecus* | *sp.* | Sichuan | Guangyuan | 105.85 | 32.44 | 1785 |
| 112 | *Rhinopithecus* | *roxellana* | guangdong | Chaozhou | 107.31 | 27.19 | 1790 |
| 113 | *Rhinopithecus* | *roxellana* | zhejiang | Chaozhou | 120.81 | 30.89 | 1790 |
| 114 | *Rhinopithecus* | *roxellana* | zhejiang | Shaoxing | 120.59 | 29.99 | 1790 |
| 115 | *Rhinopithecus* | *sp.* | guangdong | Chaozhou | 107.31 | 27.19 | 1790 |
| 116 | *Rhinopithecus* | *sp.* | zhejiang | Chaozhou | 120.81 | 30.89 | 1790 |
| 117 | *Rhinopithecus* | *sp.* | zhejiang | Shaoxing | 120.59 | 29.99 | 1790 |
| 118 | *Rhinopithecus* | *roxellana* | Chongqing | Zhongxian | 108.04 | 30.31 | 1794 |
| 119 | *Rhinopithecus* | *sp.* | Chongqing | Zhongxian | 108.04 | 30.31 | 1794 |
| 120 | *Rhinopithecus* | *roxellana* | guangdong | Jieyang | 105.49 | 26.19 | 1799 |
| 121 | *Rhinopithecus* | *sp.* | guangdong | Jieyang | 105.49 | 26.19 | 1799 |
| 122 | *Rhinopithecus* | *roxellana* | Guizhou | Sinan | 115.96 | 27.71 | 1802 |
| 123 | *Rhinopithecus* | *roxellana* | Guizhou | Yinjiang | 113.85 | 27.62 | 1802 |
| 124 | *Rhinopithecus* | *sp.* | Guizhou | Sinan | 115.96 | 27.71 | 1802 |
| 125 | *Rhinopithecus* | *sp.* | Guizhou | Yinjiang | 113.85 | 27.62 | 1802 |
| 126 | *Rhinopithecus* | *roxellana* | sichuan | Wenchuna | 103.59 | 31.47 | 1805 |
| 127 | *Rhinopithecus* | *sp.* | sichuan | Wenchuna | 103.59 | 31.47 | 1805 |
| 128 | *Rhinopithecus* | *sp.* | Sichuan | Wenchuna | 103.6 | 31.48 | 1805 |
| 129 | *Rhinopithecus* | *roxellana* | Chongqing | Rongchang | 105.61 | 29.41 | 1810 |
| 130 | *Rhinopithecus* | *roxellana* | Chongqing | Rongchang | 105.61 | 29.41 | 1810 |
| 131 | *Rhinopithecus* | *roxellana* | chonqing | Qijiang | 106.92 | 28.96 | 1810 |
| 132 | *Rhinopithecus* | *roxellana* | sichuan | Dayi | 103.26 | 30.57 | 1810 |
| 133 | *Rhinopithecus* | *roxellana* | Sichuan | Fushun | 104.97 | 29.18 | 1810 |
| 134 | *Rhinopithecus* | *roxellana* | Sichuan | Gaoxian | 104.51 | 28.43 | 1810 |
| 135 | *Rhinopithecus* | *roxellana* | sichuan | Longyuan | 105.48 | 31.92 | 1810 |
| 136 | *Rhinopithecus* | *roxellana* | Sichuan | Mabian | 103.55 | 28.84 | 1810 |
| 137 | *Rhinopithecus* | *roxellana* | sichuan | Maoxian | 103.85 | 31.68 | 1810 |
| 138 | *Rhinopithecus* | *roxellana* | Sichuan | Meigu | 103.13 | 28.32 | 1810 |
| 139 | *Rhinopithecus* | *roxellana* | Sichuan | Nanxi | 104.97 | 28.85 | 1810 |
| 140 | *Rhinopithecus* | *roxellana* | Sichuan | Pingshan | 104.15 | 28.65 | 1810 |
| 141 | *Rhinopithecus* | *roxellana* | sichuan | Qionglai | 103.23 | 30.24 | 1810 |
| 142 | *Rhinopithecus* | *roxellana* | Sichuan | XIngwen | 105.24 | 28.31 | 1810 |
| 143 | *Rhinopithecus* | *roxellana* | Sichuan | Yibin | 104.64 | 28.75 | 1810 |
| 144 | *Rhinopithecus* | *sp.* | Chongqing | Rongchang | 105.61 | 29.41 | 1810 |
| 145 | *Rhinopithecus* | *sp.* | Chongqing | Rongchang | 105.61 | 29.41 | 1810 |
| 146 | *Rhinopithecus* | *sp.* | chonqing | Qijiang | 106.92 | 28.96 | 1810 |
| 147 | *Rhinopithecus* | *sp.* | sichuan | Dayi | 103.26 | 30.57 | 1810 |
| 148 | *Rhinopithecus* | *sp.* | Sichuan | Fushun | 104.97 | 29.18 | 1810 |
| 149 | *Rhinopithecus* | *sp.* | Sichuan | Gaoxian | 104.51 | 28.43 | 1810 |
| 150 | *Rhinopithecus* | *sp.* | sichuan | Longyuan | 105.48 | 31.92 | 1810 |
| 151 | *Rhinopithecus* | *sp.* | Sichuan | Mabian | 103.55 | 28.84 | 1810 |
| 152 | *Rhinopithecus* | *sp.* | sichuan | Maoxian | 103.85 | 31.68 | 1810 |
| 153 | *Rhinopithecus* | *sp.* | Sichuan | Meigu | 103.13 | 28.32 | 1810 |
| 154 | *Rhinopithecus* | *sp.* | Sichuan | Nanxi | 104.97 | 28.85 | 1810 |
| 155 | *Rhinopithecus* | *sp.* | Sichuan | Pingshan | 104.15 | 28.65 | 1810 |
| 156 | *Rhinopithecus* | *sp.* | sichuan | Qionglai | 103.23 | 30.24 | 1810 |
| 157 | *Rhinopithecus* | *sp.* | Sichuan | XIngwen | 105.24 | 28.31 | 1810 |
| 158 | *Rhinopithecus* | *sp.* | Sichuan | Yibin | 104.64 | 28.75 | 1810 |
| 159 | *Rhinopithecus* | *sp.* | Anhui | Huangshan | 118.35 | 29.72 | 1815 |
| 160 | *Rhinopithecus* | *sp.* | Anhui | Nanling | 118.34 | 30.92 | 1815 |
| 161 | *Rhinopithecus* | *sp.* | Anhui | Xuancheng | 118.77 | 30.95 | 1815 |
| 162 | *Rhinopithecus* | *roxellana* | Sichuan | Guangan | 106.63 | 30.46 | 1816 |
| 163 | *Rhinopithecus* | *roxellana* | Sichuan | Lingshui | 106.93 | 30.33 | 1816 |
| 164 | *Rhinopithecus* | *roxellana* | Sichuan | Nanchong | 106.11 | 30.83 | 1816 |
| 165 | *Rhinopithecus* | *roxellana* | Sichuan | Pengan | 106.41 | 31.02 | 1816 |
| 166 | *Rhinopithecus* | *roxellana* | Sichuan | Xichong | 105.91 | 30.99 | 1816 |
| 167 | *Rhinopithecus* | *roxellana* | Sichuan | Yilong | 106.31 | 31.26 | 1816 |
| 168 | *Rhinopithecus* | *roxellana* | Sichuan | Yingshan | 106.57 | 31.07 | 1816 |
| 169 | *Rhinopithecus* | *roxellana* | Sichuan | Yuechi | 106.44 | 30.54 | 1816 |
| 170 | *Rhinopithecus* | *sp.* | Sichuan | Guangan | 106.63 | 30.46 | 1816 |
| 171 | *Rhinopithecus* | *sp.* | Sichuan | Leshan | 103.77 | 29.56 | 1816 |
| 172 | *Rhinopithecus* | *sp.* | Sichuan | Liangshan | 102.27 | 27.89 | 1816 |
| 173 | *Rhinopithecus* | *sp.* | Sichuan | Lingshui | 106.93 | 30.33 | 1816 |
| 174 | *Rhinopithecus* | *sp.* | Sichuan | Nanchong | 106.11 | 30.83 | 1816 |
| 175 | *Rhinopithecus* | *sp.* | Sichuan | Neijiang | 105.06 | 29.59 | 1816 |
| 176 | *Rhinopithecus* | *sp.* | Sichuan | Pengan | 106.41 | 31.02 | 1816 |
| 177 | *Rhinopithecus* | *sp.* | Sichuan | Xichong | 105.91 | 30.99 | 1816 |
| 178 | *Rhinopithecus* | *sp.* | Sichuan | Yilong | 106.31 | 31.26 | 1816 |
| 179 | *Rhinopithecus* | *sp.* | Sichuan | Yibin | 104.65 | 28.76 | 1816 |
| 180 | *Rhinopithecus* | *sp.* | Sichuan | Yingshan | 106.57 | 31.07 | 1816 |
| 181 | *Rhinopithecus* | *sp.* | Sichuan | Yuechi | 106.44 | 30.54 | 1816 |
| 182 | *Rhinopithecus* | *sp.* | Sichuan | Zigong | 104.78 | 29.35 | 1816 |
| 183 | *Rhinopithecus* | *sp.* | Sichuan | Qionglai | 103.47 | 30.42 | 1818 |
| 184 | *Rhinopithecus* | *sp.* | Gansu | Pinliang | 106.67 | 35.55 | 1819 |
| 185 | *Rhinopithecus* | *sp.* | Guangdong | Enpin | 112.31 | 22.19 | 1825 |
| 186 | *Rhinopithecus* | *roxellana* | guangdong | Zhaoqing | 104.29 | 26.86 | 1833 |
| 187 | *Rhinopithecus* | *sp.* | Guangdong | Forshan | 113.13 | 23.03 | 1833 |
| 188 | *Rhinopithecus* | *sp.* | Guangdong | Heshan | 112.96 | 22.77 | 1833 |
| 189 | *Rhinopithecus* | *sp.* | Guangdong | Yangjiang | 111.99 | 21.86 | 1833 |
| 190 | *Rhinopithecus* | *sp.* | Guangdong | Yunfu | 112.05 | 22.92 | 1833 |
| 191 | *Rhinopithecus* | *sp.* | guangdong | Zhaoqing | 104.29 | 26.86 | 1833 |
| 192 | *Rhinopithecus* | *sp.* | Guangdong | Zhaoqing | 112.47 | 23.05 | 1833 |
| 193 | *Rhinopithecus* | *sp.* | Sichuan | Beichuan | 104.36 | 31.96 | 1834 |
| 194 | *Rhinopithecus* | *sp.* | Shaanxi | Xixiang | 107.77 | 32.99 | 1835 |
| 195 | *Rhinopithecus* | *roxellana* | Guizhou | Chishui | 105.69 | 28.59 | 1841 |
| 196 | *Rhinopithecus* | *roxellana* | Guizhou | Meitan | 107.46 | 27.74 | 1841 |
| 197 | *Rhinopithecus* | *roxellana* | Guizhou | Huaireng | 106.35 | 27.75 | 1841 |
| 198 | *Rhinopithecus* | *roxellana* | Guizhou | Tongzi | 106.83 | 28.13 | 1841 |
| 199 | *Rhinopithecus* | *roxellana* | Guizhou | Xishui | 106.14 | 28.16 | 1841 |
| 200 | *Rhinopithecus* | *roxellana* | Guizhou | Zhengan | 107.44 | 28.55 | 1841 |
| 201 | *Rhinopithecus* | *sp.* | Guizhou | Cen Gong | 108.82 | 27.18 | 1841 |
| 202 | *Rhinopithecus* | *sp.* | Guizhou | Chishui | 105.69 | 28.59 | 1841 |
| 203 | *Rhinopithecus* | *sp.* | Guizhou | Meitan | 107.46 | 27.74 | 1841 |
| 204 | *Rhinopithecus* | *sp.* | Guizhou | Huaireng | 106.35 | 27.75 | 1841 |
| 205 | *Rhinopithecus* | *sp.* | Guizhou | Tongzi | 106.83 | 28.13 | 1841 |
| 206 | *Rhinopithecus* | *sp.* | Guizhou | Xishui | 106.14 | 28.16 | 1841 |
| 207 | *Rhinopithecus* | *sp.* | Guizhou | Yuping | 108.92 | 27.25 | 1841 |
| 208 | *Rhinopithecus* | *sp.* | Guizhou | Zhengan | 107.44 | 28.55 | 1841 |
| 209 | *Rhinopithecus* | *sp.* | Guizhou | Zunyi | 106.93 | 27.73 | 1841 |
| 210 | *Rhinopithecus* | *sp.* | Chongqing | Shizhu | 108.12 | 30.01 | 1843 |
| 211 | *Rhinopithecus* | *roxellana* | chongqing | Bishan | 106.23 | 29.59 | 1844 |
| 212 | *Rhinopithecus* | *roxellana* | chongqing | Dazu | 105.78 | 29.48 | 1844 |
| 213 | *Rhinopithecus* | *roxellana* | chongqing | Hechuan | 106.27 | 29.97 | 1844 |
| 214 | *Rhinopithecus* | *roxellana* | chongqing | Jiangbei | 106.57 | 29.61 | 1844 |
| 215 | *Rhinopithecus* | *roxellana* | chongqing | Jiangjin | 106.26 | 29.29 | 1844 |
| 216 | *Rhinopithecus* | *roxellana* | chongqing | Rongchang | 105.61 | 29.42 | 1844 |
| 217 | *Rhinopithecus* | *roxellana* | chongqing | Changshou | 107.08 | 29.86 | 1844 |
| 218 | *Rhinopithecus* | *roxellana* | Sichuan | Wushen | 106.29 | 30.35 | 1844 |
| 219 | *Rhinopithecus* | *sp.* | chongqing | Bishan | 106.23 | 29.59 | 1844 |
| 220 | *Rhinopithecus* | *sp.* | Chongqing | Chenkou | 108.67 | 31.95 | 1844 |
| 221 | *Rhinopithecus* | *sp.* | chongqing | Dazu | 105.78 | 29.48 | 1844 |
| 222 | *Rhinopithecus* | *sp.* | chongqing | Hechuan | 106.27 | 29.97 | 1844 |
| 223 | *Rhinopithecus* | *sp.* | chongqing | Jiangbei | 106.57 | 29.61 | 1844 |
| 224 | *Rhinopithecus* | *sp.* | chongqing | Jiangjin | 106.26 | 29.29 | 1844 |
| 225 | *Rhinopithecus* | *sp.* | chongqing | Rongchang | 105.61 | 29.42 | 1844 |
| 226 | *Rhinopithecus* | *sp.* | chongqing | Changshou | 107.08 | 29.86 | 1844 |
| 227 | *Rhinopithecus* | *sp.* | Chongqing | Chongqing | 106.56 | 29.57 | 1844 |
| 228 | *Rhinopithecus* | *sp.* | Sichuan | Wushen | 106.29 | 30.35 | 1844 |
| 229 | *Rhinopithecus* | *sp.* | Sichuan | Mianning | 102.18 | 28.56 | 1857 |
| 230 | *Rhinopithecus* | *sp.* | Sichuan | Panzhihua | 101.73 | 26.59 | 1859 |
| 231 | *Rhinopithecus* | *sp.* | Sichuan | Xichang | 102.27 | 27.9 | 1859 |
| 232 | *Rhinopithecus* | *sp.* | Jiangxi | Pingxiang | 113.86 | 27.63 | 1860 |
| 233 | *Rhinopithecus* | *sp.* | Jiangxi | Yichun | 114.42 | 27.82 | 1860 |
| 234 | *Rhinopithecus* | *sp.* | Chongqing | Qijiang | 106.66 | 29.03 | 1863 |
| 235 | *Rhinopithecus* | *sp.* | Hubei | Yichang | 111.29 | 30.7 | 1864 |
| 236 | *Rhinopithecus* | *roxellana* | hubei | Zhushan | 114.81 | 26.64 | 1865 |
| 237 | *Rhinopithecus* | *sp.* | Gansu | Minxina | 104.04 | 34.44 | 1865 |
| 238 | *Rhinopithecus* | *sp.* | hubei | Zhushan | 114.81 | 26.64 | 1865 |
| 239 | *Rhinopithecus* | *sp.* | Hubei | Zhushan | 110.24 | 32.23 | 1865 |
| 240 | *Rhinopithecus* | *sp.* | Zhejiang | Taishun | 119.72 | 27.56 | 1865 |
| 241 | *Rhinopithecus* | *sp.* | Hubei | Badong | 110.35 | 31.05 | 1866 |
| 242 | *Rhinopithecus* | *sp.* | Hubei | Yichang | 111.29 | 30.7 | 1866 |
| 243 | *Rhinopithecus* | *sp.* | Hunan | SHimen | 111.38 | 29.58 | 1868 |
| 244 | *Rhinopithecus* | *roxellana* | sichuan | Baoxing | 102.81 | 30.37 | 1870 |
| 245 | *Rhinopithecus* | *roxellana* | sichuan | Huili | 102.24 | 26.65 | 1870 |
| 246 | *Rhinopithecus* | *sp.* | sichuan | Baoxing | 102.81 | 30.37 | 1870 |
| 247 | *Rhinopithecus* | *sp.* | sichuan | Huili | 102.24 | 26.65 | 1870 |
| 248 | *Rhinopithecus* | *sp.* | Shanxi | Yangcheng | 112.42 | 35.49 | 1874 |
| 249 | *Rhinopithecus* | *sp.* | Sichuan | Huili | 102.25 | 26.66 | 1874 |
| 250 | *Rhinopithecus* | *sp.* | Chongqing | Pengshui | 108.17 | 29.3 | 1875 |
| 251 | *Rhinopithecus* | *sp.* | Guangdong | Heyuan | 114.71 | 23.75 | 1877 |
| 252 | *Rhinopithecus* | *sp.* | Guangdong | Huizhou | 114.42 | 23.12 | 1877 |
| 253 | *Rhinopithecus* | *sp.* | Guangdong | Shanwei | 115.38 | 22.79 | 1877 |
| 254 | *Rhinopithecus* | *sp.* | Guangdong | Shaoguan | 113.6 | 24.82 | 1877 |
| 255 | *Rhinopithecus* | *sp.* | Zhejiang | Taishun | 119.72 | 27.56 | 1878 |
| 256 | *Rhinopithecus* | *sp.* | Guangdong | Guangzhou | 113.27 | 23.14 | 1879 |
| 257 | *Rhinopithecus* | *sp.* | Guangdong | Jiangmen | 113.09 | 22.58 | 1879 |
| 258 | *Rhinopithecus* | *sp.* | Guangdong | Longmen | 114.26 | 23.73 | 1879 |
| 259 | *Rhinopithecus* | *sp.* | Guangdong | Shenzhen | 114.06 | 22.55 | 1879 |
| 260 | *Rhinopithecus* | *sp.* | Guangdong | Zhongshan | 113.4 | 22.52 | 1879 |
| 261 | *Rhinopithecus* | *sp.* | Guangdong | Zhongshan | 113.4 | 22.52 | 1879 |
| 262 | *Rhinopithecus* | *sp.* | Guangdong | Zhuhai | 113.58 | 22.28 | 1879 |
| 263 | *Rhinopithecus* | *sp.* | Hong Kong | Xianggang | 114.17 | 22.28 | 1879 |
| 264 | *Rhinopithecus* | *sp.* | Macau | Macau | 114.07 | 22.54 | 1879 |
| 265 | *Rhinopithecus* | *sp.* | Sichuan | Qinchengshan | 103.61 | 30.89 | 1879 |
| 266 | *Rhinopithecus* | *sp.* | Hubei | Badong | 110.35 | 31.05 | 1880 |
| 267 | *Rhinopithecus* | *roxellana* | zhejiang | Leqin | 120.98 | 28.11 | 1881 |
| 268 | *Rhinopithecus* | *sp.* | zhejiang | Leqin | 120.98 | 28.11 | 1881 |
| 269 | *Rhinopithecus* | *sp.* | Hubei | Hefeng | 110.04 | 29.9 | 1882 |
| 270 | *Rhinopithecus* | *sp.* | Shaanxi | Forpin | 108 | 33.53 | 1883 |
| 271 | *Rhinopithecus* | *sp.* | Shaanxi | Mianxian | 106.68 | 33.16 | 1883 |
| 272 | *Rhinopithecus* | *sp.* | Hubei | XIngshan | 110.75 | 31.35 | 1885 |
| 273 | *Rhinopithecus* | *roxellana* | shaanxi | Longshan | 106.21 | 35.65 | 1887 |
| 274 | *Rhinopithecus* | *roxellana* | shaanxi | Qingling | 108.33 | 33.89 | 1887 |
| 275 | *Rhinopithecus* | *sp.* | shaanxi | Longshan | 106.21 | 35.65 | 1887 |
| 276 | *Rhinopithecus* | *sp.* | shaanxi | Qingling | 108.33 | 33.89 | 1887 |
| 277 | *Rhinopithecus* | *sp.* | Shaanxi | Ningqiang | 106.26 | 32.84 | 1888 |
| 278 | *Rhinopithecus* | *roxellana* | Guizhou | Dejiang | 117.32 | 29.15 | 1890 |
| 279 | *Rhinopithecus* | *roxellana* | Guizhou | Fenggang | 107.72 | 27.95 | 1890 |
| 280 | *Rhinopithecus* | *roxellana* | Guizhou | Shiqian | 108.22 | 27.51 | 1890 |
| 281 | *Rhinopithecus* | *roxellana* | Guizhou | Songtao | 109.28 | 27.86 | 1890 |
| 282 | *Rhinopithecus* | *roxellana* | Guizhou | Suiyang | 107.19 | 27.95 | 1890 |
| 283 | *Rhinopithecus* | *roxellana* | Guizhou | Tongren | 107.39 | 27.57 | 1890 |
| 284 | *Rhinopithecus* | *roxellana* | Guizhou | Wuchuna | 114.68 | 25.95 | 1890 |
| 285 | *Rhinopithecus* | *roxellana* | Guizhou | Yanhe | 108.51 | 28.55 | 1890 |
| 286 | *Rhinopithecus* | *roxellana* | Guizhou | Zhongyi | 105.91 | 26.59 | 1890 |
| 287 | *Rhinopithecus* | *sp.* | Guizhou | Dejiang | 117.32 | 29.15 | 1890 |
| 288 | *Rhinopithecus* | *sp.* | Guizhou | Fenggang | 107.72 | 27.95 | 1890 |
| 289 | *Rhinopithecus* | *sp.* | Guizhou | Shiqian | 108.22 | 27.51 | 1890 |
| 290 | *Rhinopithecus* | *sp.* | Guizhou | Songtao | 109.28 | 27.86 | 1890 |
| 291 | *Rhinopithecus* | *sp.* | Guizhou | Suiyang | 107.19 | 27.95 | 1890 |
| 292 | *Rhinopithecus* | *sp.* | Guizhou | Tongren | 107.39 | 27.57 | 1890 |
| 293 | *Rhinopithecus* | *sp.* | Guizhou | Wuchuna | 114.68 | 25.95 | 1890 |
| 294 | *Rhinopithecus* | *sp.* | Guizhou | Yanhe | 108.51 | 28.55 | 1890 |
| 295 | *Rhinopithecus* | *sp.* | Guizhou | Zhongyi | 105.91 | 26.59 | 1890 |
| 296 | *Rhinopithecus* | *roxellana* | guangxi | Debao | 107.36 | 26.82 | 1892 |
| 297 | *Rhinopithecus* | *roxellana* | sichuan | Leba | 106.66 | 32.26 | 1893 |
| 298 | *Rhinopithecus* | *sp.* | Guangdong | Chaozhou | 116.63 | 23.66 | 1893 |
| 299 | *Rhinopithecus* | *sp.* | Guangdong | Jieyang | 116.38 | 23.56 | 1893 |
| 300 | *Rhinopithecus* | *sp.* | Guangdong | Meizhou | 116.13 | 24.29 | 1893 |
| 301 | *Rhinopithecus* | *sp.* | Guangdong | Shantou | 116.69 | 23.36 | 1893 |
| 302 | *Rhinopithecus* | *sp.* | sichuan | Leba | 106.66 | 32.26 | 1893 |
| 303 | *Rhinopithecus* | *sp.* | Sichuan | Leibo | 103.58 | 28.27 | 1893 |
| 304 | *Rhinopithecus* | *sp.* | Sichuan | Leshan | 103.77 | 29.56 | 1895 |
| 305 | *Rhinopithecus* | *sp.* | Sichuan | Liangshan | 102.27 | 27.89 | 1895 |
| 306 | *Rhinopithecus* | *sp.* | Sichuan | Neijiang | 105.06 | 29.59 | 1895 |
| 307 | *Rhinopithecus* | *sp.* | Sichuan | Yibin | 104.65 | 28.76 | 1895 |
| 308 | *Rhinopithecus* | *sp.* | Sichuan | Zigong | 104.78 | 29.35 | 1895 |
| 309 | *Rhinopithecus* | *sp.* | Guizhou | Fuquan | 107.53 | 26.69 | 1900 |
| 310 | *Rhinopithecus* | *roxellana* | shaanxi | Shiquan | 108.25 | 33.04 | 1903 |
| 311 | *Rhinopithecus* | *sp.* | shaanxi | Shiquan | 108.25 | 33.04 | 1903 |
| 312 | *Rhinopithecus* | *roxellana* | sichuan | Songpan | 103.59 | 32.63 | 1912 |
| 313 | *Rhinopithecus* | *sp.* | sichuan | Songpan | 103.59 | 32.63 | 1912 |
| 314 | *Rhinopithecus* | *sp.* | Zhejiang | Leqin | 120.99 | 28.12 | 1912 |
| 315 | *Rhinopithecus* | *sp.* | Zhejiang | Tiantai | 121.01 | 29.15 | 1915 |
| 316 | *Rhinopithecus* | *sp.* | Zhejiang | Jiande | 119.29 | 29.48 | 1919 |
| 317 | *Rhinopithecus* | *roxellana* | hubei | Yichang | 115.13 | 28.61 | 1921 |
| 318 | *Rhinopithecus* | *sp.* | hubei | Yichang | 115.13 | 28.61 | 1921 |
| 319 | *Rhinopithecus* | *sp.* | Sichuan | Songpan | 103.61 | 32.66 | 1924 |
| 320 | *Rhinopithecus* | *roxellana* | shaanxi | Zhouzhi | 108.22 | 34.16 | 1925 |
| 321 | *Rhinopithecus* | *sp.* | shaanxi | Zhouzhi | 108.22 | 34.16 | 1925 |
| 322 | *Rhinopithecus* | *sp.* | Shaanxi | Zhouzhi | 108.23 | 34.17 | 1925 |
| 323 | *Rhinopithecus* | *sp.* | Chongqing | Nanchuan | 107.11 | 29.16 | 1926 |
| 324 | *Rhinopithecus* | *sp.* | Fujian | Yongchun | 118.3 | 25.33 | 1927 |
| 325 | *Rhinopithecus* | *roxellana* | sichuan | Kangdin | 101.96 | 30.54 | 1929 |
| 326 | *Rhinopithecus* | *sp.* | Guizhou | Tongzi | 106.83 | 28.14 | 1929 |
| 327 | *Rhinopithecus* | *sp.* | Sichuan | Hejiang | 105.84 | 28.82 | 1929 |
| 328 | *Rhinopithecus* | *sp.* | sichuan | Kangdin | 101.96 | 30.54 | 1929 |
| 329 | *Rhinopithecus* | *sp.* | Sichuan | Mingshan | 103.12 | 30.08 | 1930 |
| 330 | *Rhinopithecus* | *sp.* | Chongqing | Nanchuan | 107.11 | 29.16 | 1931 |
| 331 | *Rhinopithecus* | *sp.* | Guangdong | Lechang | 113.35 | 25.14 | 1931 |
| 332 | *Rhinopithecus* | *sp.* | Sichuan | Xuanhan | 107.73 | 31.36 | 1931 |
| 333 | *Rhinopithecus* | *sp.* | Sichuan | Beichuan | 104.36 | 31.96 | 1932 |
| 334 | *Rhinopithecus* | *sp.* | Sichuan | Wanyuan | 108.04 | 32.09 | 1932 |
| 335 | *Rhinopithecus* | *roxellana* | guangxi | Fusui | 107.77 | 27.64 | 1933 |
| 336 | *Rhinopithecus* | *sp.* | Guangdong | Jieyang | 116.38 | 23.56 | 1933 |
| 337 | *Rhinopithecus* | *sp.* | Guangdong | Meizhou | 116.13 | 24.29 | 1933 |
| 338 | *Rhinopithecus* | *sp.* | Guangdong | Shantou | 116.69 | 23.36 | 1933 |
| 339 | *Rhinopithecus* | *sp.* | Shanxi | Lingchuan | 113.29 | 35.78 | 1933 |
| 340 | *Rhinopithecus* | *sp.* | Sichuan | Dujiangyan | 103.65 | 30.99 | 1933 |
| 341 | *Rhinopithecus* | *sp.* | Guangdong | Enpin | 112.31 | 22.19 | 1934 |
| 342 | *Rhinopithecus* | *sp.* | Shaanxi | XI'an | 108.95 | 34.35 | 1934 |
| 343 | *Rhinopithecus* | *roxellana* | Gansu | Kangxian | 105.79 | 32.97 | 1936 |
| 344 | *Rhinopithecus* | *sp.* | Gansu | Gannan | 102.92 | 34.99 | 1936 |
| 345 | *Rhinopithecus* | *sp.* | Gansu | Kangxian | 105.62 | 33.34 | 1936 |
| 346 | *Rhinopithecus* | *sp.* | Gansu | Kangxian | 105.79 | 32.97 | 1936 |
| 347 | *Rhinopithecus* | *sp.* | Shaanxi | XI'an | 108.95 | 34.35 | 1937 |
| 348 | *Rhinopithecus* | *sp.* | Sichuan | Luxina | 105.39 | 29.16 | 1938 |
| 349 | *Rhinopithecus* | *roxellana* | sichuan | Wenchuna | 103.59 | 31.47 | 1944 |
| 350 | *Rhinopithecus* | *sp.* | sichuan | Wenchuna | 103.59 | 31.47 | 1944 |
| 351 | *Rhinopithecus* | *sp.* | Sichuan | Wenchuna | 103.6 | 31.48 | 1944 |
| 352 | *Rhinopithecus* | *sp.* | Sichuan | Wenchuna | 103.6 | 31.48 | 1945 |
| 353 | *Rhinopithecus* | *sp.* | Guizhou | Qiandongnan | 107.99 | 26.59 | 1948 |
| 354 | *Rhinopithecus* | *sp.* | Hubei | Shien | 109.49 | 30.28 | 1966 |
| 355 | *Rhinopithecus* | *sp.* | Guizhou | Jiangkou | 108.85 | 27.71 | 1967 |
| 356 | *Rhinopithecus* | *sp.* | Guizhou | Jiangkou | 108.85 | 27.71 | 1970 |
| 357 | *Rhinopithecus* | *roxellana* | chongqing | Chenkou | 108.66 | 32.01 | 1974 |
| 358 | *Rhinopithecus* | *sp.* | chongqing | Chenkou | 108.66 | 32.01 | 1974 |
| 359 | *Rhinopithecus* | *sp.* | Sichuan | Muli | 101.29 | 27.93 | 1985 |
| 360 | *Rhinopithecus* | *sp.* | Chongqing | Nanchuan | 107.11 | 29.16 | 1991 |
| 361 | *Rhinopithecus* | *sp.* | Jiangxi | Shangyu | 114.56 | 25.79 | 1992 |
| 362 | *Rhinopithecus* | *sp.* | Guangdong | Chaozhou | 116.63 | 23.66 | 1993 |
| 363 | *Rhinopithecus* | *sp.* | Sichuan | Aba | 101.71 | 32.91 | 1993 |
| 364 | *Rhinopithecus* | *sp.* | Chongqing | Wulong | 107.77 | 29.33 | 1994 |
| 365 | *Rhinopithecus* | *sp.* | Chongqing | Chongqing | 106.56 | 29.57 | 1994 |
| 366 | *Rhinopithecus* | *sp.* | Sichuan | Jinchuan | 102.07 | 31.48 | 1994 |
| 367 | *Rhinopithecus* | *sp.* | Sichuan | Maerkang | 102.21 | 31.91 | 1995 |
| 368 | *Rhinopithecus* | *sp.* | Chongqing | Pengshui | 108.17 | 29.3 | 1997 |
| 369 | *Rhinopithecus* | *sp.* | Sichuan | Ganzi | 101.97 | 30.06 | 1997 |
| 370 | *Rhinopithecus* | *sp.* | Sichuan | Lixian | 103.17 | 31.44 | 1997 |
| 371 | *Rhinopithecus* | *sp.* | Sichuan | Luzhou | 105.45 | 28.88 | 1998 |
| 372 | *Rhinopithecus* | *sp.* | Sichuan | Xuyong | 105.45 | 28.16 | 1998 |
| 373 | *Rhinopithecus* | *sp.* | Sichuan | Jinyang | 103.25 | 27.7 | 2000 |
| 374 | *Rhinopithecus* | *sp.* | Sichuan | Batang | 99.12 | 30.01 | 2001 |
| 375 | *Rhinopithecus* | *sp.* | Gansu | Kangxian | 105.62 | 33.34 | 2002 |
| 376 | *Rhinopithecus* | *sp.* | Gansu | Longnan | 104.93 | 33.41 | 2002 |
| 377 | *Rhinopithecus* | *sp.* | Gansu | Wenxian | 104.69 | 32.95 | 2002 |
| 378 | *Rhinopithecus* | *sp.* | Gansu | Zhouqu | 104.38 | 33.79 | 2002 |
| 379 | *Rhinopithecus* | *sp.* | Guizhou | Fanjinshan | 108.77 | 27.84 | 2002 |
| 380 | *Rhinopithecus* | *sp.* | Shaanxi | Baoji | 107.24 | 34.37 | 2002 |
| 381 | *Rhinopithecus* | *sp.* | Shaanxi | Forpin | 108 | 33.53 | 2002 |
| 382 | *Rhinopithecus* | *sp.* | Shaanxi | Longxian | 106.87 | 34.9 | 2002 |
| 383 | *Rhinopithecus* | *sp.* | Shaanxi | Meixian | 107.76 | 34.28 | 2002 |
| 384 | *Rhinopithecus* | *sp.* | Shaanxi | Ningqiang | 106.26 | 32.84 | 2002 |
| 385 | *Rhinopithecus* | *sp.* | Shaanxi | Ningshan | 108.32 | 33.32 | 2002 |
| 386 | *Rhinopithecus* | *sp.* | Shaanxi | Shiquan | 108.25 | 33.04 | 2002 |
| 387 | *Rhinopithecus* | *sp.* | Shaanxi | Taibai | 107.33 | 34.06 | 2002 |
| 388 | *Rhinopithecus* | *sp.* | Shaanxi | Xixiang | 107.77 | 32.99 | 2002 |
| 389 | *Rhinopithecus* | *sp.* | Shaanxi | Yangxian | 107.55 | 33.23 | 2002 |
| 390 | *Rhinopithecus* | *sp.* | Shaanxi | Zhouzhi | 108.23 | 34.17 | 2002 |
| 391 | *Rhinopithecus* | *sp.* | Sichuan | Anxian | 104.57 | 31.54 | 2002 |
| 392 | *Rhinopithecus* | *sp.* | Sichuan | Baoxing | 102.82 | 30.37 | 2002 |
| 393 | *Rhinopithecus* | *sp.* | Sichuan | Beichuan | 104.36 | 31.96 | 2002 |
| 394 | *Rhinopithecus* | *sp.* | Sichuan | Chongzhong | 103.68 | 30.64 | 2002 |
| 395 | *Rhinopithecus* | *sp.* | Sichuan | Dayi | 103.53 | 30.59 | 2002 |
| 396 | *Rhinopithecus* | *sp.* | Sichuan | Derong | 99.29 | 28.72 | 2002 |
| 397 | *Rhinopithecus* | *sp.* | Sichuan | Dujiangyan | 103.65 | 30.99 | 2002 |
| 398 | *Rhinopithecus* | *sp.* | Sichuan | Ebian | 103.27 | 29.24 | 2002 |
| 399 | *Rhinopithecus* | *sp.* | Sichuan | Hanyuan | 102.66 | 29.35 | 2002 |
| 400 | *Rhinopithecus* | *sp.* | Sichuan | Heishui | 103 | 32.07 | 2002 |
| 401 | *Rhinopithecus* | *sp.* | Sichuan | Hongyuan | 102.55 | 32.8 | 2002 |
| 402 | *Rhinopithecus* | *sp.* | Sichuan | Hongya | 103.38 | 29.91 | 2002 |
| 403 | *Rhinopithecus* | *sp.* | Sichuan | Jiuzhaigou | 104.25 | 33.26 | 2002 |
| 404 | *Rhinopithecus* | *sp.* | Sichuan | Kangdin | 101.96 | 30 | 2002 |
| 405 | *Rhinopithecus* | *sp.* | Sichuan | Litang | 100.28 | 30 | 2002 |
| 406 | *Rhinopithecus* | *sp.* | Sichuan | Lushan | 102.93 | 30.15 | 2002 |
| 407 | *Rhinopithecus* | *sp.* | Sichuan | Ludin | 102.24 | 29.92 | 2002 |
| 408 | *Rhinopithecus* | *sp.* | Sichuan | Mabian | 103.55 | 28.84 | 2002 |
| 409 | *Rhinopithecus* | *sp.* | Sichuan | Maoxian | 103.86 | 31.69 | 2002 |
| 410 | *Rhinopithecus* | *sp.* | Sichuan | Meigu | 103.14 | 28.33 | 2002 |
| 411 | *Rhinopithecus* | *sp.* | Sichuan | Mianzhu | 104.23 | 31.34 | 2002 |
| 412 | *Rhinopithecus* | *sp.* | Sichuan | Mianning | 102.18 | 28.56 | 2002 |
| 413 | *Rhinopithecus* | *sp.* | Sichuan | Pengzhou | 103.97 | 31 | 2002 |
| 414 | *Rhinopithecus* | *sp.* | Sichuan | Pinwu | 104.54 | 32.41 | 2002 |
| 415 | *Rhinopithecus* | *sp.* | Sichuan | Qinchuanxian | 105.25 | 32.58 | 2002 |
| 416 | *Rhinopithecus* | *sp.* | Sichuan | Qionglai | 103.47 | 30.42 | 2002 |
| 417 | *Rhinopithecus* | *sp.* | Sichuan | Ruoergai | 102.97 | 33.58 | 2002 |
| 418 | *Rhinopithecus* | *sp.* | Sichuan | Shifang | 104.17 | 31.13 | 2002 |
| 419 | *Rhinopithecus* | *sp.* | Sichuan | Songpan | 103.61 | 32.66 | 2002 |
| 420 | *Rhinopithecus* | *sp.* | Sichuan | Tianquan | 102.76 | 30.07 | 2002 |
| 421 | *Rhinopithecus* | *sp.* | Sichuan | Wenchuna | 103.6 | 31.48 | 2002 |
| 422 | *Rhinopithecus* | *sp.* | Sichuan | YIngjing | 102.85 | 29.8 | 2002 |
| 423 | *Rhinopithecus* | *sp.* | Guizhou | Shiqian | 108.23 | 27.52 | 2003 |
| 424 | *Rhinopithecus* | *sp.* | Jiangxi | Anfu | 114.63 | 27.4 | 2003 |
| 425 | *Rhinopithecus* | *sp.* | Hubei | Badong | 110.35 | 31.05 | 2004 |
| 426 | *Rhinopithecus* | *sp.* | Hubei | Fangxian | 110.75 | 32.06 | 2004 |
| 427 | *Rhinopithecus* | *sp.* | Hubei | Shengnongjia | 110.68 | 31.75 | 2004 |
| 428 | *Rhinopithecus* | *sp.* | Hubei | XIngshan | 110.75 | 31.35 | 2004 |
| 429 | *Rhinopithecus* | *sp.* | Fubei | BD01 | 110.41 | 31.38 | 2020 |
| 430 | *Rhinopithecus* | *sp.* | Fubei | BD02 | 110.4 | 31.36 | 2020 |
| 431 | *Rhinopithecus* | *sp.* | Fubei | BD03 | 110.39 | 31.4 | 2020 |
| 432 | *Rhinopithecus* | *sp.* | Fubei | SNJ01 | 110.32 | 31.57 | 2020 |
| 433 | *Rhinopithecus* | *sp.* | Fubei | SNJ03 | 110.32 | 31.55 | 2020 |
| 434 | *Rhinopithecus* | *sp.* | Fubei | SNJ04 | 110.31 | 31.52 | 2020 |
| 435 | *Rhinopithecus* | *sp.* | Fubei | SNJ05 | 110.33 | 31.48 | 2020 |
| 436 | *Rhinopithecus* | *sp.* | Fubei | SNJ06 | 110.33 | 31.45 | 2020 |
| 437 | *Rhinopithecus* | *sp.* | Fubei | SNJ07 | 110.32 | 31.39 | 2020 |
| 438 | *Rhinopithecus* | *sp.* | Fubei | SNJ08 | 110.4 | 31.4 | 2020 |
| 439 | *Rhinopithecus* | *sp.* | Fubei | SNJ09 | 110.38 | 31.37 | 2020 |
| 440 | *Rhinopithecus* | *sp.* | Guizhou | Jiangkou | 108.85 | 27.71 | 2020 |
| 441 | *Rhinopithecus* | *sp.* | Guizhou | Songtao | 109.21 | 28.16 | 2020 |
| 442 | *Rhinopithecus* | *sp.* | Guizhou | Yinjiang | 108.42 | 28 | 2020 |
| 443 | *Rhinopithecus* | *sp.* | Shaanxi -Gansu | BS01 | 105.14 | 32.94 | 2020 |
| 444 | *Rhinopithecus* | *sp.* | Shaanxi -Gansu | BS02 | 105.19 | 32.91 | 2020 |
| 445 | *Rhinopithecus* | *sp.* | Shaanxi -Gansu | BS03 | 104.43 | 32.84 | 2020 |
| 446 | *Rhinopithecus* | *sp.* | Shaanxi -Gansu | BS04 | 104.49 | 32.79 | 2020 |
| 447 | *Rhinopithecus* | *sp.* | Shaanxi -Gansu | BS05 | 104.56 | 32.8 | 2020 |
| 448 | *Rhinopithecus* | *sp.* | Shaanxi -Gansu | BS06 | 104.64 | 32.8 | 2020 |
| 449 | *Rhinopithecus* | *sp.* | Shaanxi -Gansu | BS07 | 104.7 | 32.78 | 2020 |
| 450 | *Rhinopithecus* | *sp.* | Shaanxi -Gansu | BS08 | 104.7 | 32.7 | 2020 |
| 451 | *Rhinopithecus* | *sp.* | Shaanxi -Gansu | BS09 | 104.72 | 32.73 | 2020 |
| 452 | *Rhinopithecus* | *sp.* | Shaanxi -Gansu | BS10 | 104.78 | 32.68 | 2020 |
| 453 | *Rhinopithecus* | *sp.* | Shaanxi -Gansu | BS11 | 104.81 | 32.68 | 2020 |
| 454 | *Rhinopithecus* | *sp.* | Shaanxi -Gansu | BS12 | 104.88 | 32.65 | 2020 |
| 455 | *Rhinopithecus* | *sp.* | Shaanxi -Gansu | BS13 | 104.94 | 32.67 | 2020 |
| 456 | *Rhinopithecus* | *sp.* | Shaanxi -Gansu | BS14 | 104.92 | 32.63 | 2020 |
| 457 | *Rhinopithecus* | *sp.* | Shaanxi -Gansu | CQ01 | 107.5 | 33.66 | 2020 |
| 458 | *Rhinopithecus* | *sp.* | Shaanxi -Gansu | CQ02 | 107.64 | 33.67 | 2020 |
| 459 | *Rhinopithecus* | *sp.* | Shaanxi -Gansu | CQ03 | 107.62 | 33.63 | 2020 |
| 460 | *Rhinopithecus* | *sp.* | Shaanxi -Gansu | CQ04 | 107.71 | 33.64 | 2020 |
| 461 | *Rhinopithecus* | *sp.* | Shaanxi -Gansu | CQ05 | 107.69 | 33.61 | 2020 |
| 462 | *Rhinopithecus* | *sp.* | Shaanxi -Gansu | CQ06 | 107.71 | 33.54 | 2020 |
| 463 | *Rhinopithecus* | *sp.* | Shaanxi -Gansu | FP01 | 107.8 | 33.59 | 2020 |
| 464 | *Rhinopithecus* | *sp.* | Shaanxi -Gansu | FP02 | 107.92 | 33.54 | 2020 |
| 465 | *Rhinopithecus* | *sp.* | Shaanxi -Gansu | FP03 | 107.91 | 33.55 | 2020 |
| 466 | *Rhinopithecus* | *sp.* | Shaanxi -Gansu | FP04 | 107.87 | 33.69 | 2020 |
| 467 | *Rhinopithecus* | *sp.* | Shaanxi -Gansu | FP05 | 107.81 | 33.66 | 2020 |
| 468 | *Rhinopithecus* | *sp.* | Shaanxi -Gansu | FP06 | 107.78 | 33.63 | 2020 |
| 469 | *Rhinopithecus* | *sp.* | Shaanxi -Gansu | FP07 | 107.8 | 33.66 | 2020 |
| 470 | *Rhinopithecus* | *sp.* | Shaanxi -Gansu | FP08 | 107.92 | 33.54 | 2020 |
| 471 | *Rhinopithecus* | *sp.* | Shaanxi -Gansu | FP09 | 107.7 | 33.67 | 2020 |
| 472 | *Rhinopithecus* | *sp.* | Shaanxi -Gansu | GY01 | 107.89 | 33.71 | 2020 |
| 473 | *Rhinopithecus* | *sp.* | Shaanxi -Gansu | GY02 | 107.98 | 33.71 | 2020 |
| 474 | *Rhinopithecus* | *sp.* | Shaanxi -Gansu | GY03 | 107.98 | 33.68 | 2020 |
| 475 | *Rhinopithecus* | *sp.* | Shaanxi -Gansu | GY04 | 108.04 | 33.71 | 2020 |
| 476 | *Rhinopithecus* | *sp.* | Shaanxi -Gansu | GY05 | 107.91 | 33.66 | 2020 |
| 477 | *Rhinopithecus* | *sp.* | Shaanxi -Gansu | HB01 | 107.68 | 33.82 | 2020 |
| 478 | *Rhinopithecus* | *sp.* | Shaanxi -Gansu | HB02 | 107.67 | 33.81 | 2020 |
| 479 | *Rhinopithecus* | *sp.* | Shaanxi -Gansu | HB03 | 107.63 | 33.79 | 2020 |
| 480 | *Rhinopithecus* | *sp.* | Shaanxi -Gansu | HB04 | 107.6 | 33.75 | 2020 |
| 481 | *Rhinopithecus* | *sp.* | Shaanxi -Gansu | HB05 | 107.54 | 33.73 | 2020 |
| 482 | *Rhinopithecus* | *sp.* | Shaanxi -Gansu | HG01 | 108.41 | 33.72 | 2020 |
| 483 | *Rhinopithecus* | *sp.* | Shaanxi -Gansu | HG02 | 108.37 | 33.69 | 2020 |
| 484 | *Rhinopithecus* | *sp.* | Shaanxi -Gansu | HG03 | 108.38 | 33.64 | 2020 |
| 485 | *Rhinopithecus* | *sp.* | Shaanxi -Gansu | HZ01 | 107.74 | 33.86 | 2020 |
| 486 | *Rhinopithecus* | *sp.* | Shaanxi -Gansu | HZ02 | 107.89 | 33.8 | 2020 |
| 487 | *Rhinopithecus* | *sp.* | Shaanxi -Gansu | LG01 | 108.32 | 33.8 | 2020 |
| 488 | *Rhinopithecus* | *sp.* | Shaanxi -Gansu | LG02 | 108.41 | 33.86 | 2020 |
| 489 | *Rhinopithecus* | *sp.* | Shaanxi -Gansu | LX01 | 107.7 | 33.75 | 2020 |
| 490 | *Rhinopithecus* | *sp.* | Shaanxi -Gansu | LX02 | 107.79 | 33.74 | 2020 |
| 491 | *Rhinopithecus* | *sp.* | Shaanxi -Gansu | LX03 | 107.78 | 33.8 | 2020 |
| 492 | *Rhinopithecus* | *sp.* | Shaanxi -Gansu | NW01 | 107.58 | 33.78 | 2020 |
| 493 | *Rhinopithecus* | *sp.* | Shaanxi -Gansu | NW02 | 107.46 | 33.71 | 2020 |
| 494 | *Rhinopithecus* | *sp.* | Shaanxi -Gansu | NW03 | 107.4 | 33.67 | 2020 |
| 495 | *Rhinopithecus* | *sp.* | Shaanxi -Gansu | NX01 | 108.29 | 33.73 | 2020 |
| 496 | *Rhinopithecus* | *sp.* | Shaanxi -Gansu | NX02 | 108.26 | 33.61 | 2020 |
| 497 | *Rhinopithecus* | *sp.* | Shaanxi -Gansu | NX03 | 108.26 | 33.61 | 2020 |
| 498 | *Rhinopithecus* | *sp.* | Shaanxi -Gansu | PH01 | 108.46 | 33.54 | 2020 |
| 499 | *Rhinopithecus* | *sp.* | Shaanxi -Gansu | PH02 | 108.44 | 33.48 | 2020 |
| 500 | *Rhinopithecus* | *sp.* | Shaanxi -Gansu | QM01 | 105.5 | 32.91 | 2020 |
| 501 | *Rhinopithecus* | *sp.* | Shaanxi -Gansu | QM02 | 105.57 | 32.88 | 2020 |
| 502 | *Rhinopithecus* | *sp.* | Shaanxi -Gansu | TB01 | 107.72 | 34.08 | 2020 |
| 503 | *Rhinopithecus* | *sp.* | Shaanxi -Gansu | TB02 | 107.82 | 33.95 | 2020 |
| 504 | *Rhinopithecus* | *sp.* | Shaanxi -Gansu | TB03 | 107.81 | 33.88 | 2020 |
| 505 | *Rhinopithecus* | *sp.* | Shaanxi -Gansu | TB04 | 107.71 | 33.84 | 2020 |
| 506 | *Rhinopithecus* | *sp.* | Shaanxi -Gansu | TH01 | 108.09 | 33.71 | 2020 |
| 507 | *Rhinopithecus* | *sp.* | Shaanxi -Gansu | TH02 | 108.15 | 33.66 | 2020 |
| 508 | *Rhinopithecus* | *sp.* | Shaanxi -Gansu | TH03 | 108.11 | 33.64 | 2020 |
| 509 | *Rhinopithecus* | *sp.* | Shaanxi -Gansu | TH04 | 108.16 | 33.58 | 2020 |
| 510 | *Rhinopithecus* | *sp.* | Shaanxi -Gansu | TH05 | 108.1 | 33.59 | 2020 |
| 511 | *Rhinopithecus* | *sp.* | Shaanxi -Gansu | XW01 | 108.3 | 33.81 | 2020 |
| 512 | *Rhinopithecus* | *sp.* | Shaanxi -Gansu | YH01 | 105.49 | 33.1 | 2020 |
| 513 | *Rhinopithecus* | *sp.* | Shaanxi -Gansu | YH02 | 105.55 | 33.05 | 2020 |
| 514 | *Rhinopithecus* | *sp.* | Shaanxi -Gansu | YH03 | 105.63 | 32.98 | 2020 |
| 515 | *Rhinopithecus* | *sp.* | Shaanxi -Gansu | YH04 | 105.59 | 32.89 | 2020 |
| 516 | *Rhinopithecus* | *sp.* | Shaanxi -Gansu | YH05 | 105.55 | 32.91 | 2020 |
| 517 | *Rhinopithecus* | *sp.* | Shaanxi -Gansu | YH06 | 105.48 | 32.94 | 2020 |
| 518 | *Rhinopithecus* | *sp.* | Shaanxi -Gansu | YH07 | 105.42 | 32.95 | 2020 |
| 519 | *Rhinopithecus* | *sp.* | Shaanxi -Gansu | YH08 | 105.39 | 32.89 | 2020 |
| 520 | *Rhinopithecus* | *sp.* | Shaanxi -Gansu | YH09 | 105.34 | 32.88 | 2020 |
| 521 | *Rhinopithecus* | *sp.* | Shaanxi -Gansu | YH10 | 105.34 | 32.92 | 2020 |
| 522 | *Rhinopithecus* | *sp.* | Shaanxi -Gansu | YH11 | 105.35 | 32.94 | 2020 |
| 523 | *Rhinopithecus* | *sp.* | Shaanxi -Gansu | YH12 | 105.38 | 32.92 | 2020 |
| 524 | *Rhinopithecus* | *sp.* | Shaanxi -Gansu | ZZ01 | 107.85 | 33.74 | 2020 |
| 525 | *Rhinopithecus* | *sp.* | Shaanxi -Gansu | ZZ02 | 108.05 | 33.79 | 2020 |
| 526 | *Rhinopithecus* | *sp.* | Shaanxi -Gansu | ZZ03 | 108.12 | 33.77 | 2020 |
| 527 | *Rhinopithecus* | *sp.* | Shaanxi -Gansu | ZZ04 | 108.18 | 33.79 | 2020 |
| 528 | *Rhinopithecus* | *sp.* | Shaanxi -Gansu | ZZ05 | 108.26 | 33.81 | 2020 |
| 529 | *Rhinopithecus* | *sp.* | Shaanxi -Gansu | ZZ06 | 108.22 | 33.76 | 2020 |
| 530 | *Rhinopithecus* | *sp.* | Shaanxi -Gansu | ZZ07 | 108.16 | 33.74 | 2020 |
| 531 | *Rhinopithecus* | *sp.* | Shaanxi -Gansu | ZZ08 | 108.04 | 33.75 | 2020 |
| 532 | *Rhinopithecus* | *sp.* | Shaanxi -Gansu | ZZ09 | 107.91 | 33.75 | 2020 |
| 533 | *Rhinopithecus* | *sp.* | Sichuan | AZH01 | 103.2 | 30.77 | 2020 |
| 534 | *Rhinopithecus* | *sp.* | Sichuan | AZH02 | 103.19 | 30.77 | 2020 |
| 535 | *Rhinopithecus* | *sp.* | Sichuan | AZH03 | 103.19 | 30.79 | 2020 |
| 536 | *Rhinopithecus* | *sp.* | Sichuan | BDG01 | 104.01 | 31.7 | 2020 |
| 537 | *Rhinopithecus* | *sp.* | Sichuan | BDG02 | 104.16 | 31.73 | 2020 |
| 538 | *Rhinopithecus* | *sp.* | Sichuan | BDG03 | 103.91 | 31.86 | 2020 |
| 539 | *Rhinopithecus* | *sp.* | Sichuan | BH01 | 104.13 | 33.23 | 2020 |
| 540 | *Rhinopithecus* | *sp.* | Sichuan | BH02 | 104.13 | 33.3 | 2020 |
| 541 | *Rhinopithecus* | *sp.* | Sichuan | BH03 | 104.1 | 33.31 | 2020 |
| 542 | *Rhinopithecus* | *sp.* | Sichuan | BH04 | 104.07 | 33.33 | 2020 |
| 543 | *Rhinopithecus* | *sp.* | Sichuan | BSH01 | 103.68 | 31.22 | 2020 |
| 544 | *Rhinopithecus* | *sp.* | Sichuan | BSH02 | 103.72 | 31.23 | 2020 |
| 545 | *Rhinopithecus* | *sp.* | Sichuan | BSH03 | 103.75 | 31.25 | 2020 |
| 546 | *Rhinopithecus* | *sp.* | Sichuan | BY01 | 103.9 | 32.36 | 2020 |
| 547 | *Rhinopithecus* | *sp.* | Sichuan | BY02 | 103.88 | 32.24 | 2020 |
| 548 | *Rhinopithecus* | *sp.* | Sichuan | CP01 | 103.34 | 31.17 | 2020 |
| 549 | *Rhinopithecus* | *sp.* | Sichuan | CP02 | 103.55 | 31.19 | 2020 |
| 550 | *Rhinopithecus* | *sp.* | Sichuan | CP03 | 103.13 | 31.15 | 2020 |
| 551 | *Rhinopithecus* | *sp.* | Sichuan | DYG01 | 105.03 | 32.64 | 2020 |
| 552 | *Rhinopithecus* | *sp.* | Sichuan | DYG02 | 104.89 | 32.56 | 2020 |
| 553 | *Rhinopithecus* | *sp.* | Sichuan | FTZ01 | 102.88 | 30.63 | 2020 |
| 554 | *Rhinopithecus* | *sp.* | Sichuan | FTZ02 | 102.73 | 30.67 | 2020 |
| 555 | *Rhinopithecus* | *sp.* | Sichuan | FTZ03 | 102.41 | 30.86 | 2020 |
| 556 | *Rhinopithecus* | *sp.* | Sichuan | FTZ04 | 102.68 | 30.61 | 2020 |
| 557 | *Rhinopithecus* | *sp.* | Sichuan | FTZ05 | 102.76 | 30.8 | 2020 |
| 558 | *Rhinopithecus* | *sp.* | Sichuan | HL01 | 104.2 | 32.26 | 2020 |
| 559 | *Rhinopithecus* | *sp.* | Sichuan | HSH01 | 103.2 | 30.7 | 2020 |
| 560 | *Rhinopithecus* | *sp.* | Sichuan | HSH02 | 103.15 | 30.71 | 2020 |
| 561 | *Rhinopithecus* | *sp.* | Sichuan | JDS01 | 104.07 | 31.35 | 2020 |
| 562 | *Rhinopithecus* | *sp.* | Sichuan | JDS02 | 103.91 | 31.4 | 2020 |
| 563 | *Rhinopithecus* | *sp.* | Sichuan | JDS03 | 103.9 | 31.53 | 2020 |
| 564 | *Rhinopithecus* | *sp.* | Sichuan | JTKY01 | 102.37 | 30.63 | 2020 |
| 565 | *Rhinopithecus* | *sp.* | Sichuan | JTKY02 | 102.45 | 30.66 | 2020 |
| 566 | *Rhinopithecus* | *sp.* | Sichuan | JZG01 | 103.97 | 33.23 | 2020 |
| 567 | *Rhinopithecus* | *sp.* | Sichuan | LBH01 | 102.51 | 30.1 | 2020 |
| 568 | *Rhinopithecus* | *sp.* | Sichuan | LD01 | 102.34 | 29.78 | 2020 |
| 569 | *Rhinopithecus* | *sp.* | Sichuan | LDS01 | 104.08 | 32.75 | 2020 |
| 570 | *Rhinopithecus* | *sp.* | Sichuan | LHG01 | 104.72 | 32.53 | 2020 |
| 571 | *Rhinopithecus* | *sp.* | Sichuan | LHG02 | 104.69 | 32.52 | 2020 |
| 572 | *Rhinopithecus* | *sp.* | Sichuan | LS01 | 102.98 | 30.5 | 2020 |
| 573 | *Rhinopithecus* | *sp.* | Sichuan | LS02 | 103.03 | 30.54 | 2020 |
| 574 | *Rhinopithecus* | *sp.* | Sichuan | LXHK01 | 103.63 | 31.27 | 2020 |
| 575 | *Rhinopithecus* | *sp.* | Sichuan | LXHK02 | 103.64 | 31.22 | 2020 |
| 576 | *Rhinopithecus* | *sp.* | Sichuan | LXHK03 | 103.58 | 31.16 | 2020 |
| 577 | *Rhinopithecus* | *sp.* | Sichuan | LXHK04 | 103.6 | 31.17 | 2020 |
| 578 | *Rhinopithecus* | *sp.* | Sichuan | MYL01 | 103.27 | 31.44 | 2020 |
| 579 | *Rhinopithecus* | *sp.* | Sichuan | MYL02 | 103.31 | 31.44 | 2020 |
| 580 | *Rhinopithecus* | *sp.* | Sichuan | MZ01 | 105.43 | 32.92 | 2020 |
| 581 | *Rhinopithecus* | *sp.* | Sichuan | MZ02 | 105.47 | 32.87 | 2020 |
| 582 | *Rhinopithecus* | *sp.* | Sichuan | MZ03 | 105.48 | 32.91 | 2020 |
| 583 | *Rhinopithecus* | *sp.* | Sichuan | PK01 | 104.11 | 32.1 | 2020 |
| 584 | *Rhinopithecus* | *sp.* | Sichuan | PK02 | 104.09 | 32.05 | 2020 |
| 585 | *Rhinopithecus* | *sp.* | Sichuan | QFS01 | 104.21 | 31.74 | 2020 |
| 586 | *Rhinopithecus* | *sp.* | Sichuan | QFS02 | 104.24 | 31.75 | 2020 |
| 587 | *Rhinopithecus* | *sp.* | Sichuan | QFS03 | 104.29 | 31.75 | 2020 |
| 588 | *Rhinopithecus* | *sp.* | Sichuan | QFS04 | 104.24 | 31.76 | 2020 |
| 589 | *Rhinopithecus* | *sp.* | Sichuan | SDG01 | 103.09 | 32.28 | 2020 |
| 590 | *Rhinopithecus* | *sp.* | Sichuan | SDG02 | 102.87 | 32.25 | 2020 |
| 591 | *Rhinopithecus* | *sp.* | Sichuan | SGNS01 | 102.78 | 31.07 | 2020 |
| 592 | *Rhinopithecus* | *sp.* | Sichuan | SGNS02 | 102.7 | 31.03 | 2020 |
| 593 | *Rhinopithecus* | *sp.* | Sichuan | SGNS03 | 102.61 | 31.05 | 2020 |
| 594 | *Rhinopithecus* | *sp.* | Sichuan | TJH01 | 104.84 | 32.62 | 2020 |
| 595 | *Rhinopithecus* | *sp.* | Sichuan | TJH02 | 104.81 | 32.64 | 2020 |
| 596 | *Rhinopithecus* | *sp.* | Sichuan | TJH03 | 104.78 | 32.61 | 2020 |
| 597 | *Rhinopithecus* | *sp.* | Sichuan | TJH04 | 104.7 | 32.63 | 2020 |
| 598 | *Rhinopithecus* | *sp.* | Sichuan | TJH05 | 104.65 | 32.81 | 2020 |
| 599 | *Rhinopithecus* | *sp.* | Sichuan | WJ01 | 104.27 | 32.93 | 2020 |
| 600 | *Rhinopithecus* | *sp.* | Sichuan | WL01 | 103.16 | 31 | 2020 |
| 601 | *Rhinopithecus* | *sp.* | Sichuan | WL01 | 104.45 | 32.12 | 2020 |
| 602 | *Rhinopithecus* | *sp.* | Sichuan | WL02 | 103.24 | 31.07 | 2020 |
| 603 | *Rhinopithecus* | *sp.* | Sichuan | WL02 | 104.27 | 32.22 | 2020 |
| 604 | *Rhinopithecus* | *sp.* | Sichuan | WL03 | 103.35 | 31.16 | 2020 |
| 605 | *Rhinopithecus* | *sp.* | Sichuan | WL03 | 104.15 | 32.32 | 2020 |
| 606 | *Rhinopithecus* | *sp.* | Sichuan | WL04 | 103.19 | 31.07 | 2020 |
| 607 | *Rhinopithecus* | *sp.* | Sichuan | WL04 | 104.98 | 32.54 | 2020 |
| 608 | *Rhinopithecus* | *sp.* | Sichuan | XBD01 | 103.92 | 32.51 | 2020 |
| 609 | *Rhinopithecus* | *sp.* | Sichuan | XBD02 | 104.01 | 32.45 | 2020 |
| 610 | *Rhinopithecus* | *sp.* | Sichuan | XBD03 | 103.97 | 32.53 | 2020 |
| 611 | *Rhinopithecus* | *sp.* | Sichuan | XHG01 | 104.25 | 32.62 | 2020 |
| 612 | *Rhinopithecus* | *sp.* | Sichuan | XHG02 | 104.44 | 32.57 | 2020 |
| 613 | *Rhinopithecus* | *sp.* | Sichuan | XZZG01 | 103.87 | 31.98 | 2020 |
| 614 | *Rhinopithecus* | *sp.* | Sichuan | XZZG02 | 103.93 | 32.11 | 2020 |
| 615 | *Rhinopithecus* | *sp.* | Sichuan | XZZG03 | 104.05 | 32.06 | 2020 |
| 616 | *Rhinopithecus* | *sp.* | Sichuan | XZZG04 | 104.01 | 32.1 | 2020 |
| 617 | *Rhinopithecus* | *sp.* | Sichuan | YJ01 | 102.51 | 29.88 | 2020 |
| 618 | *Rhinopithecus* | *sp.* | Sichuan | YJS01 | 104.55 | 32.49 | 2020 |
| 619 | *Rhinopithecus* | *roxellana* | Guizhou | Sinan | 108.25 | 27.93 | 1800-1849 |
| 620 | *Rhinopithecus* | *roxellana* | Sichuan | Anxian | 104.6 | 31.5 | 1800-1849 |
| 621 | *Rhinopithecus* | *roxellana* | Sichuan | Daxueshan | 101.5 | 30.5 | 1800-1849 |
| 622 | *Rhinopithecus* | *roxellana* | Sichuan | Pengxian | 104.1 | 30.9 | 1800-1849 |
| 623 | *Rhinopithecus* | *roxellana* | Sichuan | Xiaoliangshan | 102.8 | 26.8 | 1800-1849 |
| 624 | *Rhinopithecus* | *roxellana* | Chongqing | Dianjiang | 107.34 | 30.33 | 1821-1850 |
| 625 | *Rhinopithecus* | *roxellana* | Chongqing | Dianjiang | 107.34 | 30.32 | 1821-1850 |
| 626 | *Rhinopithecus* | *roxellana* | Chongqing | Fengdu | 107.73 | 29.86 | 1821-1850 |
| 627 | *Rhinopithecus* | *roxellana* | Chongqing | Liangping | 107.81 | 30.67 | 1821-1850 |
| 628 | *Rhinopithecus* | *roxellana* | Chongqing | Liangping | 107.81 | 30.67 | 1821-1850 |
| 629 | *Rhinopithecus* | *roxellana* | Gansu | Wenxian | 105.42 | 32.81 | 1821-1850 |
| 630 | *Rhinopithecus* | *roxellana* | Gansu | Wudu | 104.93 | 33.39 | 1821-1850 |
| 631 | *Rhinopithecus* | *roxellana* | shaanxi | Xixiang | 107.76 | 32.98 | 1821-1850 |
| 632 | *Rhinopithecus* | *roxellana* | sichuan | Beichuan | 104.31 | 31.89 | 1821-1850 |
| 633 | *Rhinopithecus* | *roxellana* | Sichuan | Fushun | 104.97 | 29.18 | 1821-1850 |
| 634 | *Rhinopithecus* | *roxellana* | sichuan | Yuexi | 105.26 | 30.19 | 1821-1850 |
| 635 | *Rhinopithecus* | *sp.* | Chongqing | Dianjiang | 107.34 | 30.33 | 1821-1850 |
| 636 | *Rhinopithecus* | *sp.* | Chongqing | Dianjiang | 107.34 | 30.32 | 1821-1850 |
| 637 | *Rhinopithecus* | *sp.* | Chongqing | Fengdu | 107.73 | 29.86 | 1821-1850 |
| 638 | *Rhinopithecus* | *sp.* | Chongqing | Liangping | 107.81 | 30.67 | 1821-1850 |
| 639 | *Rhinopithecus* | *sp.* | Chongqing | Liangping | 107.81 | 30.67 | 1821-1850 |
| 640 | *Rhinopithecus* | *sp.* | Gansu | Wenxian | 105.42 | 32.81 | 1821-1850 |
| 641 | *Rhinopithecus* | *sp.* | Gansu | Wudu | 104.93 | 33.39 | 1821-1850 |
| 642 | *Rhinopithecus* | *sp.* | shaanxi | Xixiang | 107.76 | 32.98 | 1821-1850 |
| 643 | *Rhinopithecus* | *sp.* | sichuan | Beichuan | 104.31 | 31.89 | 1821-1850 |
| 644 | *Rhinopithecus* | *sp.* | Sichuan | Fushun | 104.97 | 29.18 | 1821-1850 |
| 645 | *Rhinopithecus* | *sp.* | sichuan | Yuexi | 105.26 | 30.19 | 1821-1850 |
| 646 | *Rhinopithecus* | *roxellana* | Guizhou | Dejiang | 108.12 | 28.26 | 1850-1899 |
| 647 | *Rhinopithecus* | *roxellana* | Guizhou | Wuchuna | 107.89 | 28.56 | 1850-1899 |
| 648 | *Rhinopithecus* | *roxellana* | Sichuan | Anxian | 104.6 | 31.5 | 1850-1899 |
| 649 | *Rhinopithecus* | *roxellana* | Sichuan | Daxueshan | 101.5 | 30.5 | 1850-1899 |
| 650 | *Rhinopithecus* | *roxellana* | Sichuan | Pengxian | 104.1 | 30.9 | 1850-1899 |
| 651 | *Rhinopithecus* | *roxellana* | Sichuan | Xiaoliangshan | 102.8 | 26.8 | 1850-1899 |
| 652 | *Rhinopithecus* | *roxellana* | Sichuan | Anxian | 104.6 | 31.5 | 1900-1949 |
| 653 | *Rhinopithecus* | *roxellana* | Sichuan | Daxueshan | 101.5 | 30.5 | 1900-1949 |
| 654 | *Rhinopithecus* | *roxellana* | Sichuan | Pengxian | 104.1 | 30.9 | 1900-1949 |
| 655 | *Rhinopithecus* | *roxellana* | Sichuan | Xiaoliangshan | 102.8 | 26.8 | 1900-1949 |
| 656 | *Rhinopithecus* | *roxellana* | Sichuan | Wanyuan | 108.03 | 32.08 | 1932-1974 |
| 657 | *Rhinopithecus* | *sp.* | Sichuan | Wanyuan | 108.03 | 32.08 | 1932-1974 |
| 658 | *Rhinopithecus* | *roxellana* | Sichuan | Anxian | 104.6 | 31.5 | 1950-1999 |
| 659 | *Rhinopithecus* | *roxellana* | Sichuan | Daxueshan | 101.5 | 30.5 | 1950-1999 |
| 660 | *Rhinopithecus* | *roxellana* | Sichuan | Pengxian | 104.1 | 30.9 | 1950-1999 |
| 661 | *Rhinopithecus* | *roxellana* | Sichuan | Xiaoliangshan | 102.8 | 26.8 | 1950-1999 |
| 662 | *Rhinopithecus* | *roxellana* | Sichuan | Fengxian | 106.52 | 33.91 | 2020-2024 |
| 663 | *Rhinopithecus* | *roxellana* | Sichuan | Liuba | 106.92 | 33.618 | 2020-2024 |
| 664 | *Rhinopithecus* | *roxellana* | Sichuan | Anxian | 104.6 | 31.5 | Before1800 |
| 665 | *Rhinopithecus* | *roxellana* | Sichuan | Daxueshan | 101.5 | 30.5 | Before1800 |
| 666 | *Rhinopithecus* | *roxellana* | Sichuan | Pengxian | 104.1 | 30.9 | Before1800 |
| 667 | *Rhinopithecus* | *roxellana* | Sichuan | Xiaoliangshan | 102.8 | 26.8 | Before1800 |

Table S3 . Fossil sites of *Homo* during Pleistocene in China, based on which Figure 9 was generated.

Table S3. Location of *Homo* fossil sites in China

| **Number** | **Genius** | **Province** | **County** | **Time** |
| --- | --- | --- | --- | --- |
| 1 | Homo | Yunnan | Chuxiong | Early Pleistocene |
| 2 | Homo | Shaanxi | Lantian | Early Pleistocene |
| 3 | Homo | Hubei | Yunyang(Yunxian) | Early Pleistocene |
| 4 | Homo | Shaanxi | Lantian | Middle Pleistocene |
| 5 | Homo | Jiangsu | Tangshan | Middle Pleistocene |
| 6 | Homo | Anhui | Hexian | Middle Pleistocene |
| 7 | Homo | Guangxi(Guangdong) | Lingshan | Late Pleistocene |
| 8 | Homo | Guangxi | Tiandong | Late Pleistocene |
| 9 | Homo (Early) | Beijing | Zhoukoudian | Middle Pleistocene |
| 10 | Homo (Early) | Shaanxi | Dali | Middle Pleistocene |
| 11 | Homo (Early) | Liaoning | Dashiqiao | Middle Pleistocene |
| 12 | Homo (Early) | Guangdong | Qujiang | Middle Pleistocene |
| 13 | Homo (Early) | Shanxi | Yanggao | Middle Pleistocene |
| 14 | Homo (Early) | Shanxi | Linfen | Late Pleistocene |
| 15 | Homo (Early) | Guizhou | Tongzi | Late Pleistocene |
| 16 | Homo (Early) | Guizhou | Puding | Late Pleistocene |
| 17 | Homo (Early) | Yunnan | Chenggong | Late Pleistocene |
| 18 | Homo (Early) | Guangxi | Chongzuo | Late Pleistocene |
| 19 | Homo (Early) | Shaanxi | Yanan | Late Pleistocene |
| 20 | Homo (Early) | Shanxi | Suoxian | Late Pleistocene |
| 21 | Homo (Early) | Henan | Xuchang | Late Pleistocene |
| 22 | Homo (Early) | Guangxi | Tiandong | Late Pleistocene |
| 23 | Homo (Early) | Guizhou | Panzhou | Middle Pleistocene |
| 24 | Homo (Early) | Shandong | Zibo | Middle Pleistocene |
| 25 | Homo (Early) | Anhui | Hefei | Middle Pleistocene |
| 26 | Homo (Early) | Shaanxi | Changwu | Late Pleistocene |
| 27 | Homo (Early) | Hubei | Changyang | Late Pleistocene |
| 28 | Homo (Early) | Hebei | Yangyuan | Early Pleistocene |
| 29 | Homo (Early) | Guangdong | Fengkai | Late Pleistocene |
| 30 | Homo (Late) | Guangxi | Liujiang | Late Pleistocene |
| 31 | Homo (Late) | Beijing | Zhoukoudian | Late Pleistocene |
| 32 | Homo (Late) | Yunnan | Lijiang | Late Pleistocene |
| 33 | Homo (Late) | Sichuan | Ziyang | Late Pleistocene |
| 34 | Homo (Late) | Mongolia | Erdos | Late Pleistocene |
| 35 | Homo (Late) | Hunan | Daoxian | Late Pleistocene |
| 36 | Homo (Late) | Henan | Zhumadian | Late Pleistocene |
| 37 | Homo (Late) | Guangxi | Pingle | Late Pleistocene |
| 38 | Homo (Late) | Guangxi | Longlin | Late Pleistocene |
| 39 | Homo (Late) | Yunnan | Mengzi | Late Pleistocene |
| 40 | Homo (Late) | Guizhou | Xingyi | Late Pleistocene |
| 41 | Homo (Late) | Taiwan | Tainan | Late Pleistocene |
| 42 | Homo (Late) | Shandong | Xintai | Late Pleistocene |
| 43 | Homo (Late) | Zhejiang | Jiande | Late Pleistocene |
| 44 | Homo (Late) | Liaoning | Jianping | Late Pleistocene |
| 45 | Homo (Late) | Jiangsu | Dantu | Late Pleistocene |
| 46 | Homo (Late) | Guangxi | Laibin | Late Pleistocene |
| 47 | Homo (Late) | Guangxi | Guiling | Late Pleistocene |
| 48 | Homo (Late) | Guangxi | Lipu | Late Pleistocene |
| 49 | Homo (Late) | Jilin | Yushu | Late Pleistocene |
| 50 | Homo (Late) | Jiangsu | Sihong | Late Pleistocene |

References

Chang, C. H., M. Takai, & S. Ogino. (2012). First discovery of colobine fossils from the early to middle Pleistocene of southern Taiwan. *Journal of Human Evolution*, *63*(3), 439-451. <https://doi.org/10.1016/j.jhevol.2012.03.005>

Colbert, E. H., & D. A. Hooijer. (1953). Pleistocene mammals from the limestone fissures of Szechuan, China. *American museum of natural history*, *102*(1024). <http://www.rhinoresourcecenter.com/pdf_files/127/1271894385.pdf>

Gu, Y. M., & C. K. Hu. (1991). A Fossil Cranium of *Rhinopithecus*  Found in Xinan, Henan Province. *Vertebrata Palasiatica*, *29*(1).

Gu, Y. M., & N. G. Jablonski. (1989). A reassessment of *Megamacaca lantianensis* of Gongwangling, Shaanxi Province. *Acta Anthropologica Sinica*, *8*(4), 343―346

Jablonski, N. G. (1998). The response of catarrhine primates to pleistocene environmental fluctuations in East Asia. *Primates*, *39*(1), 29-37. <https://doi.org/10.1007/bf02557741>

Jablonski, N. G. (2008). Fossil Old World monkeys: the late Neogene radiation. In W. C. Hartwig (Ed.), *The Primate Fossil Record.* (pp. 255-299). Cambridge University Press.

Jablonski, N. G., & Y. M. Gu. (1991). A reassessment of *Megamacaca lantianensis,* a large monkey from the Pleistocene of north-central China. *Journal of Human Evolution*, *20*(1), 51-66. <https://doi.org/10.1016/0047-2484(91)90045-w>

Jablonski, N. G., X. P. Ji, J. Kelley, L. J. Flynn, C. L. Deng, & D. F. Su. (2020). *Mesopithecus pentelicus* from Zhaotong, China, the easternmost representative of a widespread Miocene cercopithecoid species. *Journal of Human Evolution*, *146*. <https://doi.org/10.1016/j.jhevol.2020.102851>

Ji, X. P., D. Youlatos, N. G. Jablonski, R. L. Pan, C. X. Zhang, P. Li, M. Tang, T. S. Yu, W. Q. Li, C. L. Deng, & S. Li. (2020). Oldest colobine calcaneus from East Asia (Zhaotong, Yunnan, China). *Journal of Human Evolution*, *147*, 102866. <https://doi.org/10.1016/j.jhevol.2020.102866>

Kang, A., Y. Xie, J. R. Tang, E. W. Sanderson, J. R. Ginsberg, & E. Zhang. (2010). Historic distribution and recent loss of tigers in China. *Integrative Zoology*, *5*(4), 335-341. <https://doi.org/10.1111/j.1749-4877.2010.00221.x>

Kuang, W. m., D. Zinner, Y. Li, X. Q. Yao, C. Roos, & L. Yu. (2023). Recent Advances in Genetics and Genomics of Snub-Nosed Monkeys (*Rhinopithecus*) and Their Implications for Phylogeny, Conservation, and Adaptation. *Genes (Basel)*, *14*(5). <https://doi.org/10.3390/genes14050985>

Li, B. G., R. L. Pan, & C. E. Oxnard. (2002). The extinction of the golden monkey (*Rhinopithecus*) in China during the Last 400 Years. *International Journal of Primatology*, *23*(6), 1227-1244. <https://doi.org/10.1023/A:1021122819845>

Li, B. G., H. Zhang, M. Li, X. L. Jiang, P. F. Fan, J. Zhou, S. T. Guo, X. G. Qi, J. H. Li, J. Q. Lu, D. P. Xia, L. W. Cui, Z. F. Xiang, Q. H. Zhou, Z. P. Huang, C. M. Huang, W. Xiao, H. J. Hu, Z. X. Zhou, . . . R. L. Pan. (2024). Achievements and challenges of primate conservation in China. *Diversity and Conservation, Zoological Research*, *1*(1), 66-74. <https://doi.org/10.24272/j.issn.2097-3772.2023.298>

Li, Y. L., K. Huang, S. Y. Tang, L. Feng, J. Yang, Z. H. Li, & B. G. Li. (2020). Genetic Structure and Evolutionary History of *Rhinopithecus roxellana* in Qinling Mountains, Central China. *Frontiers in Genetics*, *11*, 611914. <https://doi.org/10.3389/fgene.2020.611914>

Liedigk, R., M. Yang, N. G. Jablonski, F. Momberg, T. Geissmann, N. Lwin, T. H. Hla, Z. J. Liu, B. Wong, M. Li, Y. C. Long, Y. P. Zhang, T. Nadler, D. Zinner, & C. Roos. (2012). Evolutionary history of the odd-nosed monkeys and the phylogenetic position of the newly described Myanmar snub-nosed monkey Rhinopithecus strykeri. *PLoS One*, *7*(5), e37418. <https://doi.org/10.1371/journal.pone.0037418>

Pan, H., R. Hou, H. Zhang, Y. P. Li, Z. P. Huang, L. W. Cui, & W. Xiao. (2024a). Surviving at the highest and coldest: Nutritional and chemical components of fallback foods for Yunnan snub‐nosed monkeys. *Ecology Evolution*, *14*(4), e11219. <https://doi.org/10.1002/ece3.11219>

Pan, H., X. P. Ji, D. Youlatos, Y. Chen, H. Zhang, G. G. Guo, J. Wang, K. Huang, R. Hou, G. He, S. T. Guo, P. Zhang, B. G. Li, & R. L. Pan. (2025). Morphometric Study on the Mandible of Colobine Fossil (*Mesopithecus pentelicus*) Found in East Asia, a Comparison With Extant Taxa. *American Journal of Primatology*, *87*(1), e23706. <https://doi.org/10.1002/ajp.23706>

Pan, H., H. Zhang, D. Youlatos, J. Wang, G. He, S. t. Guo, K. Huang, R. Hou, R. L. Pan, G. Fang, Y. L. Li, P. Zhang, & B. G. Li. (2024b). Evolutionary Insights from Dental Diversity in Afro-Asian Primates. *Diversity*, *16*(9). <https://doi.org/10.3390/d16090565>

Pan, Y. R., & N. G. Jablonski. (1987). The age and geographical distribution of fossil *Cercopithecids* in China [journal article]. *Human Evolution*, *2*(1), 59-69. <https://doi.org/10.1007/bf02436531>

Xiang, Z. F., S. Huo, L. Wang, L. W. Cui, W. Xiao, R. C. Quan, & Z. Tai. (2007). Distribution, status and conservation of the black-and-white snub-nosed monkey *Rhinopithecus bieti* in Tibet. *Oryx*, *41*(4), 525-531. <https://doi.org/10.1017/s0030605307012124>

Yang, M. Y., Y. Q. Yang, D. Y. Cui, G. Fickenscher, D. Zinner, C. Roos, & M. Brameier. (2012). Population genetic structure of Guizhou snub-nosed monkeys (*Rhinopithecus brelichi*) as inferred from mitochondrial control region sequences, and comparison with *R. roxellana* and *R. bieti*. *American Journal of Physical Anthropology*, *147*(1), 1-10. <https://doi.org/10.1002/ajpa.21618>

Zhang, H., J. Q. Lu, S. Y. Tang, Z. P. Huang, L. W. Cui, D. Y. Lan, H. T. Wang, H. Rong, W. Xiao, S. T. Guo, G. He, K. Huang, P. Zhang, H. Pan, C. E. Oxnard, R. L. Pan, & B. G. Li. (2022). Southwest China, the last refuge of continental primates in East Asia. *Biological Conservation*, *273*, 109681. <https://doi.org/10.1016/j.biocon.2022.109681>
